# Ancestral hydrocarbon metabolism enables PET degradation by a natural bacterial consortium

**DOI:** 10.1128/msystems.00829-26

**Published:** 2026-08-21

**Authors:** Sabrina Edwards, Danny Rice, Patricio Palomino, Irene Newton, Jay L. Mellies

**Affiliations:** 1 Biology Department, Reed College 6686, Portland, Oregon, USA; 2 Department of Biology, Indiana University 123993, Bloomington, Indiana, USA; CNRS Delegation Alpes, Villeurbanne, Rhône-Alpes, France

**Keywords:** *Pseudomonas*, *Bacillus*, polyethylene terephthalate, plastic biodegradation, adaptation, biotransformation, environmental consortia, horizontal gene transfer, proteomics

## Abstract

Plastic biodegradation in natural environments is increasingly recognized as a multi-organism process; however, the mechanisms enabling coordinated depolymerization and metabolism of polyethylene terephthalate (PET) remain poorly understood. Previously, we demonstrated that a full consortium containing three *Pseudomonas* and two *Bacillus* strains isolated from hydrocarbon-rich coastal soils of Galveston Bay, Texas, can synergistically depolymerize PET plastic and utilize it as a sole carbon source, a capacity not observed in individual isolates. In this report, using integrated comparative genomics, proteomics, and chemical analyses, we show that PET degradation in this system reflects exaptation of hydrocarbon metabolism, reinforced by metabolic division of labor. Within this naturally occurring consortium, *Bacillus* strains persist under environmental stress, establish biofilms, and perform essential secondary hydrolysis, while *Pseudomonas* strains catabolize aromatic monomers and buffer oxidative stress. Genes supporting these functions are enriched within the accessory genomes of the consortium strains, indicating consortium-enriched horizontal gene transfer. In addition to the canonical two-step hydrolytic pathway well documented in PET biodegradation, we identify a secondary methylation- and redox-associated process, mechanisms where the full consortium acts on the oligomer mono(2-hydroxyethyl) terephthalate (MHET), yielding nearly complete conversion to terephthalic acid and methylated MHET. Together, these findings support a model in which PET degradation is driven by pre-existing hydrocarbon metabolic traits distributed across the native consortium, particularly stress-response and redox pathways, aromatic catabolism, and alcohol catabolism.

**IMPORTANCE:**

Environmental plastic degradation is rarely accomplished by a single organism; however, the microbial mechanisms enabling community-level polyethylene terephthalate (PET) plastic breakdown remain poorly understood. This study shows that a bacterial consortium isolated from crude petroleum-contaminated coastal soils degrades PET by coordinating older hydrocarbon, aromatic, stress-response, and redox-associated metabolisms rather than by acquiring a dedicated PET pathway. Predicted horizontal gene transfer events were linked mainly to survival, biofilm formation, and metabolic flexibility, not PET depolymerization itself. These findings shift the focus from searching for single PET-degrading organisms toward understanding how microbial communities combine pre-existing metabolic tools to process synthetic polymers and manage the chemical stress created during biodegradation.

## INTRODUCTION

Galveston Bay, Texas, hosts extensive natural oil seeps where crude petroleum has migrated into coastal soils for millennia and has been a hotspot of anthropogenic oil spills (1–3). As a result, these chronically hydrocarbon-exposed environments are dominated by hydrocarbonoclastic bacteria (HCB), including the genera *Pseudomonas*, *Acinetobacter*, *Rhodococcus*, and *Bacillus* (4–8). Over evolutionary timescales, these taxa have refined pathways for utilizing petroleum-derived compounds (6–13). Building on this ecological context, we previously isolated a microbial consortium from Galveston Bay, composed of three *Pseudomonas* and two *Bacillus* strains that synergistically degrade both polyethylene terephthalate (PET) and polyethylene (PE) plastics (14, 15). Because synthetic plastics are synthesized from petroleum-derived monomers, we hypothesized that hydrocarbon-adapted bacteria within this consortium may be evolutionarily primed to exploit plastic-associated substrates through pre-existing hydrocarbon metabolic pathways.

PET, the most widely used packaging and textile polymer, is assembled from terephthalic acid (TPA) and ethylene glycol (EG), while PE consists of long hydrocarbon chains. Although these monomers are readily metabolized by HCB, such as this described consortium, once polymerized, these compounds form highly durable plastic polymers that persist in the environment, polluting aquatic and terrestrial ecosystems (16–20). When plastics enter the environment, abiotic weathering (UV, oxidation, and mechanical stress) gradually fractures polymer chains, introducing hydrophilic groups, increasing surface reactivity, and enabling microbial colonization (21–25). These changes increase bioavailability and allow microbes to form complex biofilms and secrete extracellular enzymes that fragment polymers into soluble oligomers and monomers, which can be converted to metabolic intermediates and subsequently funneled back into pre-existing metabolic pathways (26, 27).

To date, researchers have identified a large and growing catalog of individual PET-degrading enzymes. These include PETase from *Ideonella sakaiensis*, isolated from e-waste-associated environments, and highly active thermophilic cutinases derived from biopolymer-rich sites such as leaf compost (e.g., leaf compost cutinase, LCC) (28–30), which can either partially cleave PET to oligomers bis-(2-hydroxyethyl) terephthalate (BHET) and mono-(2-hydroxyethyl) terephthalate (MHET) or fully cleave PET to the original monomers, TPA and EG. While these enzymes demonstrate that PET hydrolysis is biochemically feasible for biotechnology recycling efforts, environmental plastic degradation is rarely attributable to a single enzyme. Instead, increasing evidence suggests that PET breakdown in natural systems reflects coordinated activity within microbial consortia, driven by metabolic specialization across community members (30–33). Despite this knowledge, the mechanisms enabling cooperative and synergistic PET degradation and assimilation in environmental consortia remain poorly resolved. It remains unclear whether these biodegradation capabilities arise primarily through horizontal gene transfer, gradual sequence refinement, or adaptation of pre-existing hydrocarbon or aromatic metabolic pathways. Although PET biodegradation has been partially characterized in several systems, the evolutionary basis of PET degradation in natural microbial communities is still not well understood (29, 34).

Previous pangenomic and transcriptomic analyses of the five-member consortium initially revealed that these isolates shared significant overlaps in accessory genomes compared to non-PET degraders, including genes implicated in the degradation of multiple bioplastics and synthetic plastics, like numerous hydrolases and oxidoreductases (35). Given that this consortium may have been evolutionarily “primed” to exploit plastic-associated substrates due to their hydrocarbon-rich origins, we sought to resolve how these functions are distributed and coordinated during PET degradation using horizontal gene transfer (HGT) (36) analysis and functional characterization of secreted proteomes during growth on PET. These data sets revealed significant inter-genus genetic sharing and complementary roles in extracellular depolymerization, redox handling, and downstream processing of PET-derived intermediates, a pattern that was further supported by the detection of methylated mono(2-hydroxyethyl) terephthalate (MMHET) during PET incubation.

Our results show that PET degradation is not the product of a single enzyme or pathway; rather, it is a community-level adaptation. This *Bacillus-Pseudomonas* consortium degrades PET through a cooperative division of labor. When strains are absent, MHET and/or TPA accumulate, which can inhibit further depolymerization. Notably, MHET catabolism does not exclusively follow canonical hydrolytic fates, suggesting the emergence of alternative, environmentally relevant chemical transformations during PET depolymerization. In this study, we examine evidence that adaptation to plastic as a substrate is consortia-based and distributed across complementary physiological roles, where survival and carbon assimilation depend on cooperation, stress buffering, and coordinated metabolic activity.

## MATERIALS AND METHODS

### Bacterial strains and bioinformatics

The five bacterial strains comprising the consortium used in this study were previously obtained from petroleum-polluted soils in Galveston Bay, Texas (37). Genomes were sequenced using hybrid Illumina/Nanopore technology and assembled with Unicycler v0.5.0 (BioProject PRJNA517285). Strains include *Bacillus thuringiensis* 9.1 and *Bacillus cereus* 13.1 (SAMN41232458 and SAMN41232461 and *Pseudomonas* species strains 9.2, 10, and 13.2 (SAMN41232459, SAMN41232460, and SAMN41232462). Integrated Microbiobial Genomes & Microbiomes (IMG/MER) of the Joint Genome Institute (JGI) data were cross-referenced with the National Center for Biotechnology Information (NCBI) GenBank/RefSeq identifiers, which served as the primary accession references for downstream analyses. Genomic, HGT, and proteomic data sets were standardized through reciprocal best-hit Basic Local Alignment Search Tool (BLAST) alignment (*E*-value ≤ 1 × 10^−^⁵). Due to near-complete genomic identity and an inability to resolve strain-specific contributions under consortium conditions, *Pseudomonas* strains 10 and 13.2 collapsed into a single operational unit for comparative analyses. Protein identifiers were curated using Batch Entrez (NCBI), mapped to UniParc identifiers, and corresponding UniParc FASTA sequences were functionally annotated using GhostKOALA (KEGG) to assign Kyoto Encyclopedia of Genes and Genomes (KEGG) Orthology (KO) identifiers and associated metabolic function.

### Horizontal gene transfer identification

HGT predictions were computed using DarkHorse v2.0 (36, 38, 39) against the NCBI nr database (August 2024). Proteins were aligned using BLASTP (-evalue 1e-3, -max_target_seqs 100000), and self-matches were excluded using the -e option. DarkHorse was run with parameters -f 0.1 -n 3 -b 0 -d 1. Candidate HGT genes were identified using DarkHorse based on lineage probability index (LPI) scores. Genes with LPI ≤0.50 and *E*-value ≤1 × 10^−^⁵ were retained as putative HGT candidates. Homologous genes were grouped by unique identifiers (UIDs; besthit_id, File S1), and occurrences across genomes were treated as independent instances to assess strain-level distribution and gene sharing. Taxonomic assignments from DarkHorse were standardized to the class level, and viral-associated candidates were retained as a separate category. Functional and independent taxonomic annotations were obtained using GhostKOALA (40) to assign KO identifiers and Biomolecular Relations in Information Transmission and Expression (BRITE) functional categories. Genes lacking KO assignments were classified as “unassigned.” Annotated and unassigned HGT-associated genes were then manually curated into PET-relevant functional categories using IMG/MER, NCBI GenBank/RefSeq, UniProt/UniParc, KEGG database entries, and supporting literature. Categories included hydrolytic/oxidative enzymes, outer membrane and aromatic transport, biofilm formation and surface interaction, stress response and detoxification, quorum sensing and regulation, central metabolism, mobile genetic elements/phage-associated functions, and unknown/other. Hydrolytic/oxidative enzymes and transport-associated genes were prioritized because of their potential roles in PET depolymerization, intermediate detoxification, and monomer uptake or assimilation.

### Bacterial growth and biodegradation assays

Bacterial strains were grown overnight in Lysogeny Broth (LB) at 37°C. The five-member consortium was inoculated to a final OD_600_ of 0.01 into 1.5-cm glass tubes containing 5 mL of liquid carbon free-basal medium (LCFBM) medium in triplicate. The LCFBM base consisted of 10 mM sodium phosphate buffer (pH 7.4) supplemented with 0.05% (wt/vol) yeast extract, 0.2% (wt/vol) (NH_4_)_2_SO_4_, and 1% (vol/vol) trace elements (0.1% [wt/vol] FeSO_4_·7H_2_O, 0.1% [wt/vol] MgSO_4_·7H_2_O, 0.01% [wt/vol] CuSO_4_·5H_2_O, 0.01% [wt/vol] MnSO_4_·5H_2_O, and 0.01% [wt/vol] ZnSO_4_·7H_2_O). For PET plastic growth, the cultures were grown in triplicate in LCFBM supplemented with 1% (wt/vol) PET granules (Sigma-Aldrich, St. Louis, MO) and grown statically for 7 weeks; growth was monitored by CFU/mL counts. For the investigation of MHET degradation, 5 mL tubes containing LCFBM were supplemented with 0.2% (wt/vol) MHET (Advanced ChemBlock Inc., Hayward, CA) and incubated aerobically for 21 days.

### Proteome extraction and analysis

Extracellular proteins from culture supernatants and PET biofilms were extracted using high-salt ECM disruption (1 M NaCl) to avoid cell lysis. Culture supernatants (25 mL) were cleared (8,000 × *g*, 10 min, 25°C), adjusted to 1 M NaCl, and incubated for 15 min. Biofilm proteins were extracted from five PET pellets by vigorous washing in 1 mL of 1 M NaCl for 15 min. Both extracts were clarified at 5,000 × *g* for 10 min. Proteins were precipitated overnight at –20°C using four volumes of cold acetone. The resulting pellets were centrifuged (13,000–15,000 × *g*, 10 min), air-dried, resuspended in 100 µL ultrapure water, and stored at –20°C. Samples were subsequently analyzed by liquid chromatography-tandem mass spectrometry (LC-MS/MS (performed at the Oregon Health & Science University (OHSU) Proteomics Core Facility. Proteins were identified by searching against specific UniProt (41) reference proteomes, *Bacillus albus* (UP000181873), *B. thuringiensis* (UP000195991), and *Pseudomonas* spp. (UP001231507 and UP000238367). Functional category distributions are shown by both detected protein count and abundance normalized to GroEL, because it is a chaperone protein. Count represents the number of detected proteins/genes assigned to each functional category, while abundance represents the summed protein abundance within each category normalized to GroEL for the corresponding strain. Functional categories were assigned using UniProt/UniParc, KEGG Orthology, IMG/MER annotations, protein families (PFAM) domain classifications, and supporting literature. To maintain conservative interpretation, the proteins were grouped into top-level functional roles rather than narrow KEGG sub-pathway or enzyme-specific classifications, particularly when annotations were broad, incomplete, or unresolved. PFAM was used to support conservative classification of proteins with broad, unresolved, or missing KEGG annotations.

### Quantification of aromatic products by high-performance liquid chromatography (HPLC)

Aromatic depolymerization products were quantified using an Agilent 1290 Infinity HPLC (Agilent Technologies, Santa Clara, CA) equipped with a diode array detector (DAD). Separation was performed on a ZORBAX Eclipse Plus C18, 100 × 2.1 mm, 1.8-µm column (Agilent Technologies, Santa Clara, CA) at 40°C. An isocratic mobile phase of water:acetonitrile (80:20) acidified with 0.1% (wt/vol) formic acid was applied at 0.450 mL/min, with UV detection at 250 nm. Both standards (TPA, MHET, and BHET) and experimental samples were solubilized in 90:10 acetonitrile:DMSO prior to injection.

### Identification of unknown metabolites (LC-MS)

To identify non-standard compounds and intermediates, culture samples were diluted in 100% acetonitrile and analyzed using a Shimadzu 8030 Triple Quadrupole LC/MS system (Shimadzu, Kyoto, Japan). Chromatographic separation was performed using the same mobile phase described above with an Agilent InfinityLab Poroshell 120 EC-C18, 4.6 × 100 mm, 4-µm column (Agilent Technologies, Santa Clara, CA). Mass spectral data were acquired in negative electrospray ionization (ESI) mode to structurally characterize aromatic byproducts and unknown intermediates generated during biodegradation. The ESI peaks of TPA and MHET standards were experimentally determined to be at 165.02 *m/z* and 209.05 *m/z*.

## RESULTS AND DISCUSSION

### Analysis of horizontal gene transfer events

The bacterial consortium possesses a large accessory genome (35) rich in diverse hydrolytic and oxidative enzymes involved in the biodegradation of plastics (28, 42, 43). As accessory genes are often transmissible, we hypothesized these functional traits may have been acquired from distant taxa (44). To test this hypothesis, we used DarkHorse (36) to detect atypical evolutionary signatures. The tool calculates a lineage probability index (LPI), where a low score indicates that a gene’s closest homologs are found in distant taxa rather than the host lineage. This identifies probable HGT events based on atypical ancestry, regardless of transfer direction.

Putative HGT candidates identified from DarkHorse were filtered by statistical significance (*E*-value ≤1 × 10^−^⁵), then by binning normalized LPI scores according to taxonomic lineage to define self-match thresholds (File S1). As self-matches were enriched at higher LPI values, with an average LPI ≥0.50, an LPI ≤0.50 threshold was applied to identify putative horizontal gene candidates. Candidates with LPI <0.20 were predominantly viral, fungal, or eukaryotic genes within all strains (Fig. S1). Eukaryotic and fungal candidates were excluded from further analysis due to apparent eukaryotic assignments likely reflecting taxonomic misannotation of contaminated Whole genome sequencing (WGS) contigs. Viral (phage-associated) hits accounted for ~13% of candidates with LPI <0.10, primarily in *Bacillus* strain 13.1 (Table S1), and were excluded due to limited metabolic relevance. Protein-coding density and predicted HGT frequencies varied but remained within ranges typical of bacterial genomes (Table 1).

**TABLE 1:** NCBI genome statistics and summary of predicted HGT candidates’ genes from the five-member consortium^*a*^

Genome statistics	*Bacillus* strains		*Pseudomonas* strains	
	9.1	13.1	9.2	10/13.2
Total protein CDS genes	5,513	6,577	5,693	5,220
DarkHorse predicted HGT genes	216	365	46	233
DarkHorse % predicted HGT	4.10%	6.80%	0.80%	4.50%

^*a*^ Counts of total coding sequences (CDSs), initial DarkHorse HGT predictions of putative horizontally acquired genes, including phage, excluding false positives within each consortium member. 10/13.2 are 99.9% identical with minor strain differences.

**Fig 1:**
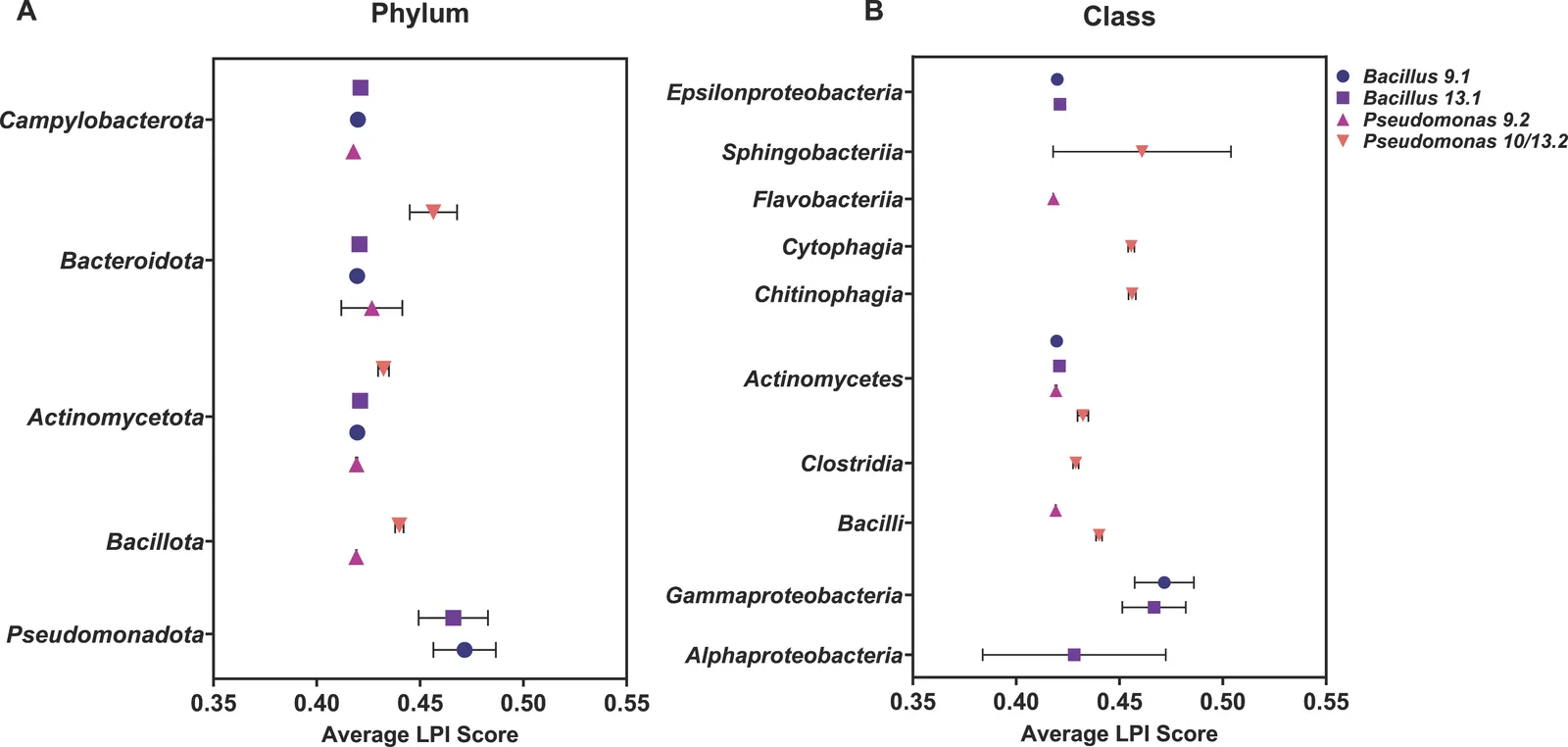
LPI scores for HGT candidates across consortium strains following filtering against self-match thresholds. Candidates below the self-match threshold (LPI ≤ 0.50) are displayed. (**A**) Filtered bacterial phylum-level candidates were retained and were phylum filtered to those with >2 instances per individual strain query. (**B**) At the class level, filtered candidates were assigned to 10 bacterial classes. Error bars indicate one standard deviation.

Because DarkHorse donor assignments rely on global compositional similarity and because publicly available sequence databases are dominated by clinically relevant taxa, not environmental taxa, predictions more frequently default to high-level matches at the phylum or class level rather than the genus or species level. Thus, all predictions were examined at the bacterial phylum and class level rather than at the species level. Within the full consortium members, five bacterial phyla (*Campylobacterota, Bacteroidota, Actinomycetota, Bacillota*, and *Pseudomonata*) were identified as putative HGT candidate taxa, with 10 unique classes represented (Fig. 1A). After using LPI scores to identify which taxa were putative HGT candidates, we examined the distribution of gene candidates among these taxa. Because single-gene predictions can reflect spurious annotations or transient genetic acquisitions, we prioritized genes with LPI scores <0.50 and from phyla that were detected multiple times ( >2) (Fig. S2) for further analysis, as recurrence suggests stable inheritance or repeated acquisition events and therefore a higher likelihood of biological relevance than single-copy occurrences (Fig. 1B).

**Fig 2:**
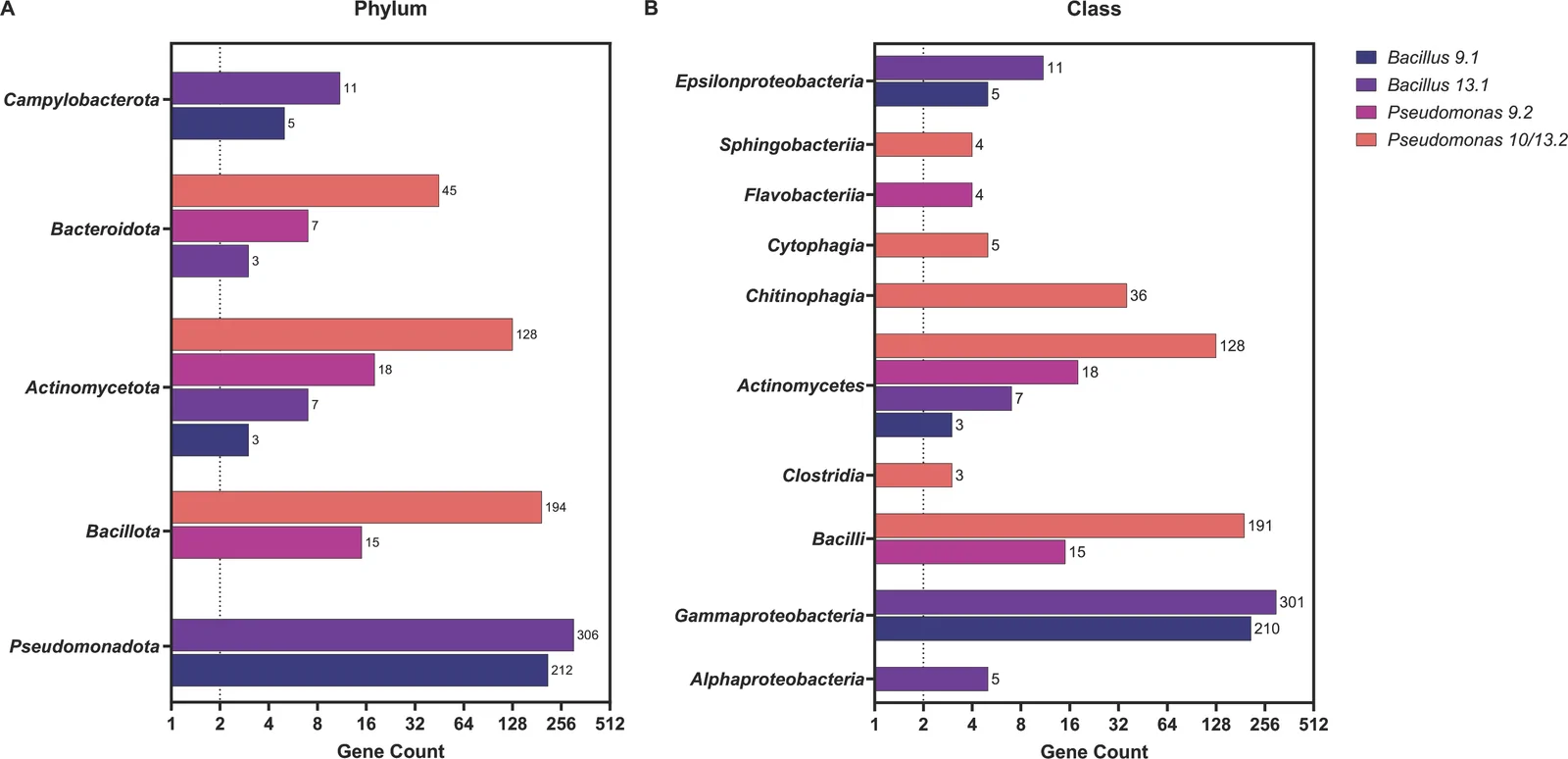
Distribution of bacterial candidate HGT genes and their inferred taxonomic origins across consortium strains using a maximum LPI threshold of ≤ 0.50. (**A**) Phylum-level distribution of inferred donor lineages, restricted to candidates meeting the LPI threshold and represented by >2 genes. (**B**) Class-level distributions reveal reciprocal enrichment patterns, with *Bacillus* strains (9.1 and 13.1) enriched in Gammaproteobacteria-associated genes (212 and 306, respectively), and *Pseudomonas* strains (9.2 and 10/13.2) enriched in *Bacilli*-associated genes (15 and 194, respectively), consistent with bidirectional cross-class gene exchange.

Illustrated in Fig. 2, HGT candidates were predominantly detected in *Pseudomonadota* at the phylum level and *Gammaproteobacteria* at the class level for the *Bacillus* strains 9.1 and 13.1. The HGT predicted genes in the *Pseudomonas* strains 10/13.2 (strains 10 and 13.2 show 99.9% DNA sequence identity) were predominantly predicted to be *Bacillota/Bacilli*-derived, with up to 191 genes detected in *Pseudomonas* strain 10/13.2. On the individual strain level, both *Bacillus* strains had highly similar HGT events (apart from multiple viral elements seen in strain 13.1 [File S1]). In contrast, *Pseudomonas* strain 9.2 differed dramatically from 10/13.2, with minimal detected HGT events of 0.8% (Table 1), and only 15 genes derived from *Bacilli* and 18 genes from *Actinomycetes* (Fig. 2). From this analysis, over 600 unique HGT candidates (*n* = 616) within consortium members were identified. These results initially indicate that most HGT genes within the consortium were assigned to two distinct taxa: *Gammaproteobacteria* and *Bacilli*, directly reflecting the taxonomic classes within the consortium. DarkHorse may correctly identify donors/recipients at a higher taxonomic level, but genus and species assignments might be inaccurate. Therefore, we performed a secondary taxonomic and functional annotation of the unique HGT candidates using GhostKOALA (40).

**Fig 3:**
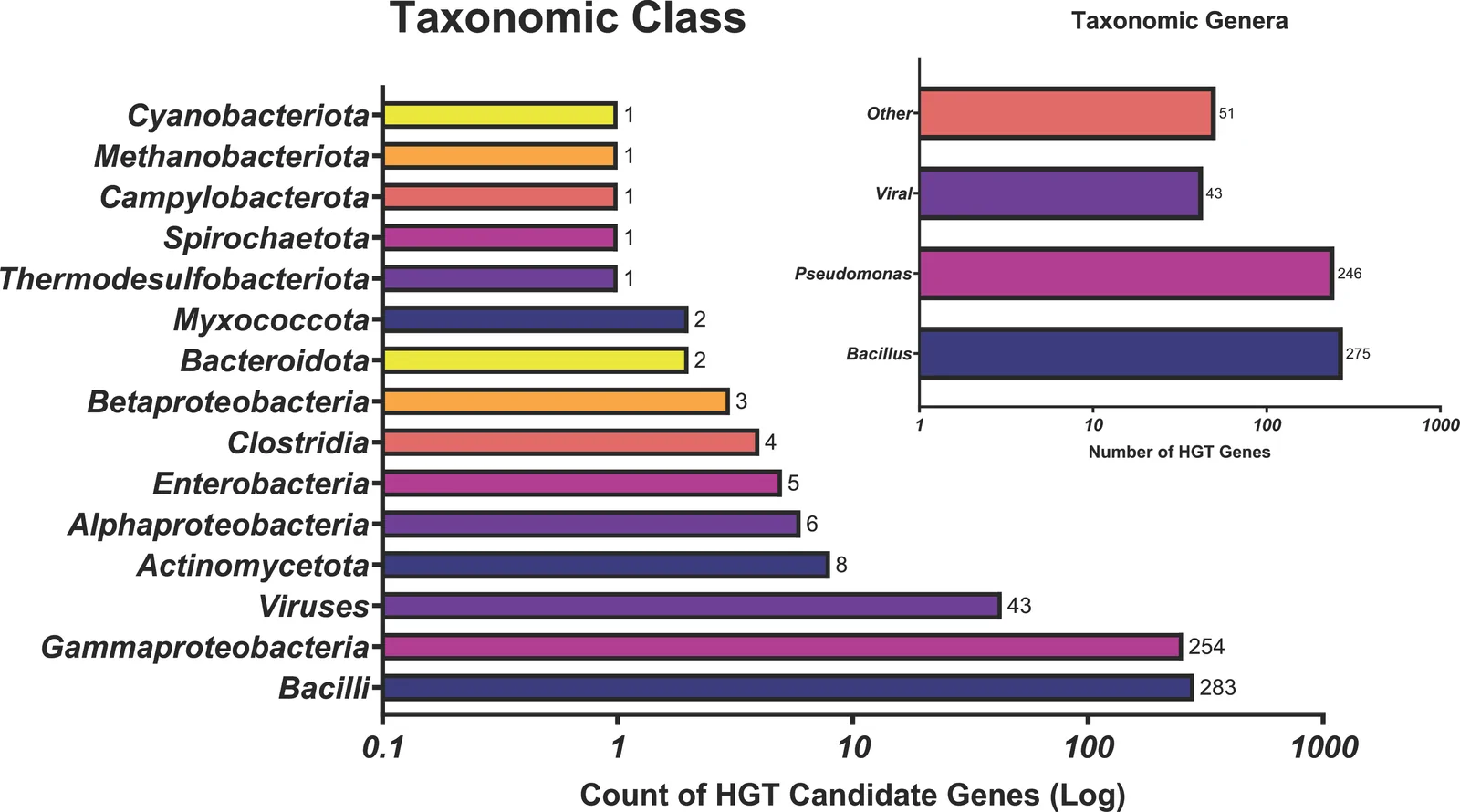
Distribution and taxonomic KEGG classification of candidate HGT genes across consortium members. Class-level distribution of HGT candidates following LPI filtering (≤0.50), displayed on a log scale. HGT candidates were predominantly assigned to *Bacilli* (*n* = 283) and *Gammaproteobacteria* (*n* = 254). Collapsed genus-level distribution highlighting dominant contributors. The majority of HGT candidates mapped to *Bacillus* (*n* = 275) and *Pseudomonas* (*n* = 246), while low-frequency taxa were grouped as “other” (*n* = 51). Virus-associated candidates (*n* = 43) mapped primarily to *Bacillus* and *Pseudomonas* phage, consistent with phage-mediated gene mobility within dominant consortium lineages.

Taxonomic sequence-based assignment of genes from HGT candidate taxa (KEGG) indicated that of the 616 genes identified through HGT analysis, 521 were assigned to either *Pseudomonas* (*n* = 246) or *Bacillus* (*n* = 275) (Fig. 3). Notably, besides *Pseudomonas* or *Bacillus*, and despite removal of phage-specific HGT candidates (File S1), most remaining HGT-associated genes were annotated as viral in origin (*n* = 43), with the majority classified as *Pseudomonas*- and *Bacillus*-associated (File S2). This enrichment of host-specific viral genes across both genera supports frequent phage-mediated gene exchange within the consortium and is consistent with cross-genera gene transfer. The shared viral associations further suggest prolonged ecological proximity and genetic interaction, likely ancestral.

**Fig 4:**
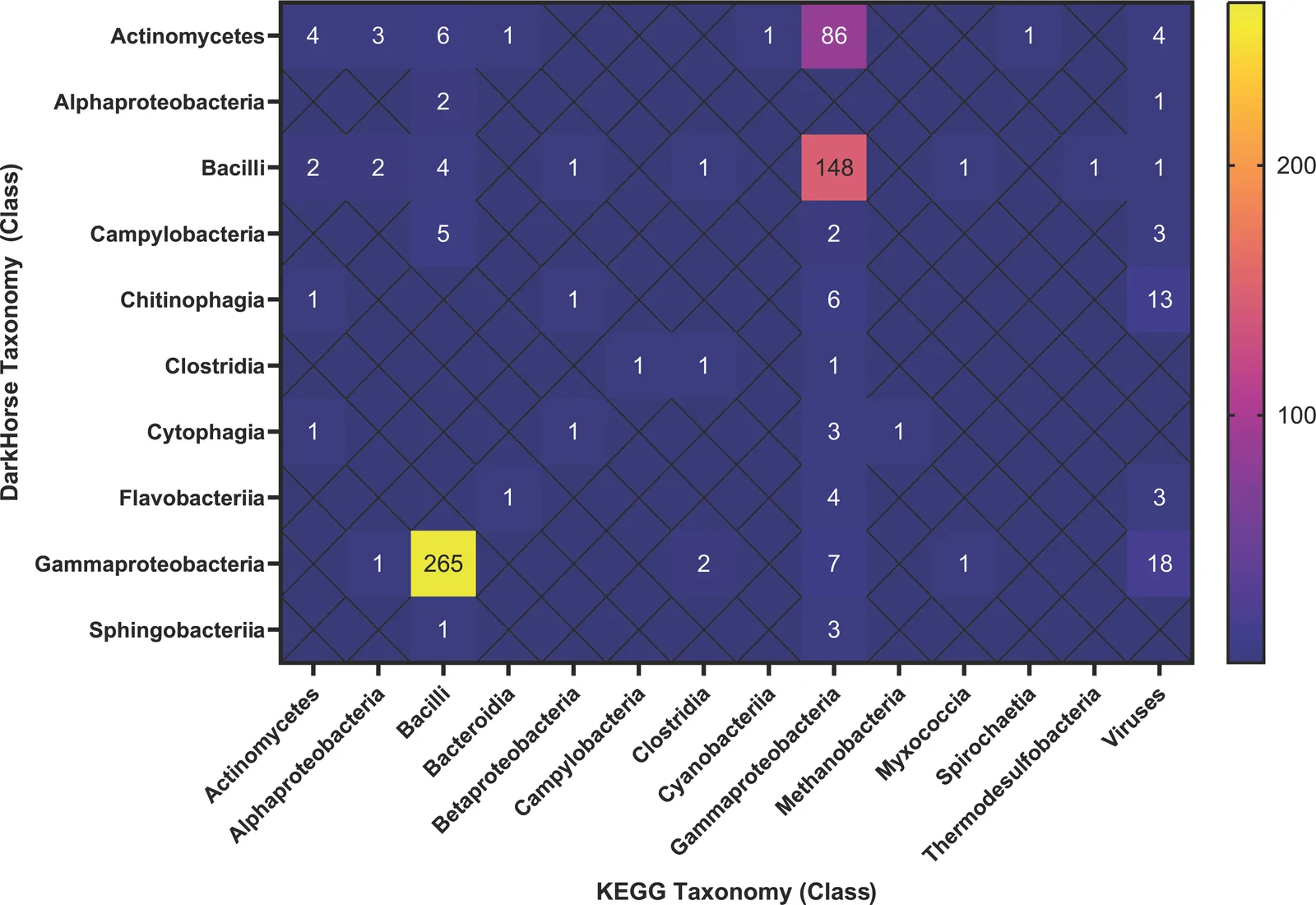
Comparison of KEGG and DarkHorse taxonomic classifications highlights structured HGT between Bacilli and Gammaproteobacteria. Heatmap comparing KEGG-derived taxonomic classifications of HGT candidate UIDs with DarkHorse lineage assignments. Values represent gene counts for each taxonomic pairing. Strong enrichment is observed between *Bacilli* and *Gammaproteobacteria*, with frequent cross-assignment between these classes. KEGG classifications more closely align with the consortium query strain (File S1) than with DarkHorse candidate lineage assignments. This discordance suggests recipient-biased lineage inference by DarkHorse and retention of donor-associated signals in sequence-based taxonomy annotated via KEGG, consistent with ancestral HGT events.

KEGG-based taxonomic classification of HGT candidates showed systematic discordance with DarkHorse lineage assignments (Fig. 4), particularly between *Bacilli* and *Gammaproteobacteria*, where genes detected in one class by DarkHorse frequently mapped to the other by KEGG. This pattern suggests DarkHorse may provide a genomic context of gene detection (putative recipient), whereas sequence-based classification retains donor- or ancestral-associated signals, although without further investigation, directionality cannot be resolved. *Actinomycetota*-associated candidates illustrate this trend. While DarkHorse assigned numerous genes to *Actinomycetota* across consortium members (Fig. 2), KEGG classified only a small subset as Actinomycetota (*n* = 8; Fig. 3), with most mapping to *Gammaproteobacteria* (Fig. 4). These patterns likely reflect possible ancestral gene exchange or shared ecological adaptation among co-occurring soil taxa rather than recent, directional transfer, as members of this phylum are ubiquitous in soil and hydrocarbon-rich environments, alongside the consortium members, and also are commonly associated with plastic degradation pathways (45, 46).

Of all HGT genes, 85% were assigned to *Pseudomonas* and *Bacillus* genera, while 96% of all functional annotations were also mapped to these two genera using KEGG functional categories (Table 2). Annotated HGT candidates were predominantly associated with core metabolic and housekeeping functions, including pathways related to amino acid, carbohydrate, and cofactor metabolism, as well as genetic information processing and cell regulation. With respect to PET biodegradation, minimal genes mapped to pathways relevant to xenobiotic biodegradation and metabolism (*n* = 4), including aromatic compound metabolism (e.g., TPA-associated pathways) (47) and broader oxidative and hydrolytic processes. Additional pathway categories of interest included energy metabolism (*n* = 5), lipid metabolism (*n* = 4), and environmental information processing (*n* = 22), which may support co-metabolic activity and substrate uptake during polymer degradation.

**TABLE 2:** Functional classification of candidate HGT genes based on KEGG annotations HGT^*d*^

KEGG functional category	Total HGT genes^*a*^ (*n*)	*Bacillus*/*Pseudomonas^b^* (*n*)	Proportion identical^*c*^ (%)
**Metabolism**	**94**	**92**	**98%**
Amino acid metabolism	13	13	100%
Carbohydrate metabolism	14	14	100%
Energy metabolism	5	5	100%
Glycan biosynthesis and metabolism	6	6	100%
Lipid metabolism	4	4	100%
Metabolism of cofactors and vitamins	20	19	95%
Metabolism of other amino acids	2	2	100%
Metabolism of terpenoids and polyketides	1	1	100%
Nucleotide metabolism	8	8	100%
Xenobiotic biodegradation and metabolism	4	4	100%
Protein families: metabolism	5	5	100%
Unclassified: metabolism	12	11	92%
**Genetic information processing**	**65**	**61**	**94%**
Genetic information processing	18	16	89%
Protein families: genetic information processing	43	41	95%
Unclassified genetic information processing	4	4	100%
**Signaling and cellular processes**	**77**	**73**	**95%**
Cellular processes	7	7	100%
Environmental information processing	22	22	100%
Protein families: signaling and cellular processes	37	33	89%
Unclassified: signaling and cellular processes	11	11	100%
**Unclassified/other**	**24**	**24**	**100%**
Human disease	2	2	100%
Unclassified	22	22	100%
**Totals KEGG annotations**	**260**	**249**	**96%**
**Totals HGT genes (unannotated)**	**616**	**521**	**85%**

^*a*^ All HGT gene candidates were grouped into hierarchical KEGG functional categories, and counts are shown.

^*b*^ All candidates were KEGG taxonomically categorized as *Bacillus *and *Pseudomonas.*

^*c*^ Percentages represent the proportion of genes within each category associated with these dominant taxa.

^*d*^ Across all functional categories, HGT candidates were predominantly *Bacillus* and *Pseudomonas *in origin (95% of annotated, 85% of all HGT candidates). Broad categories of genes are indicated by bold text.

PET-associated and unclassified genes were categorized further by functional classes of interest, including hydrolytic/oxidative enzymes (48–56) and outer-membrane transport/aromatic transport (57–64), as these genes could be potentially involved in polymer depolymerization, intermediate detoxification, and monomer transport and assimilation (Table S2). Genes linked to biofilm formation, surface interaction, and stress response were also detected, suggesting roles in substrate colonization and persistence under nutrient limitation (65–67). In *Bacillus* strains, horizontally transferred genes related to biofilm formation (e.g., *spaA*), stress tolerance, and starvation responses (e.g*., cstA*) were identified, traits that may enhance adhesion to polymer surfaces and persistence under nutrient-limited conditions. In *Pseudomonas* strains, oxidoreductases and transporters involved in core aromatic metabolism pathways were detected as HGT candidates, including the protocatechuate catabolic gene *pcaD*, as well as dehydrogenases and transcriptional regulators, all involved in downstream monomer assimilation (Table S2) (45, 68–71). However, KEGG-based classification suggested that these loci are most consistent with *Pseudomonas/Gammaproteobacteria* ancestry, despite the presence of homologs in *Actinomycetota*, a phylum commonly associated with hydrocarbon-contaminated environments. Thus, these genes may reflect shared hydrocarbon-associated metabolic history rather than recent directional transfer from *Actinomycetota*.

Many HGT candidates were not assigned to specific KEGG pathways (~42%), suggesting functional novelty or incomplete pathway annotation. Importantly, no complete genes with annotated PET depolymerization/assimilation function were identified via DarkHorse analysis. Instead, HGT appears to contribute individual genes that enhance metabolic flexibility, stress resilience, and surface/biofilm functionality, rather than serving as a primary mechanism for the dissemination of canonical plastic-degrading enzymes, such as PETases, within the consortium.

### Mobile genetic elements suggest xenobiotic specialization

Most predicted HGT events occurred within the same genus or between closely associated taxa, with enrichment between *Bacillus* and *Pseudomonas*. This pattern is consistent with inter-genus gene exchange among co-occurring lineages, likely facilitated by close spatial arrangement (e.g*.,* biofilms) in the environment (65, 72). Although HGT candidates were distributed across both *Bacillus* and *Pseudomonas* within the consortium, we next focused on *Pseudomonas* to investigate potential mechanisms of gene transfer due to their capacity to efficiently assimilate TPA, unlike their *Bacillus* counterparts. Xenobiotic metabolic pathways in *Pseudomonas*, including those involved in terephthalate degradation, are frequently associated with plasmid-borne elements (73–78). Screening for plasmids and other mobile genetic elements was conducted using geNomad (79), an AI tool that classifies MGE through the IMG/JGI portal (80).

The primary hit from this analysis was a fragmented 232 gene region encoding MobF (geNomad Score = 0.9741) within *Pseudomonas* strains 10/13.2 (File S3), with high similarity to plasmids from other *Pseudomonas* spp., including *Pseudomonas putida* S12 (ID = 0.834), a solvent-tolerant strain that can degrade styrene plastics (77, 81, 82). *Pseudomonas hunanensis* was the predicted host, isolated from manganese-polluted soil in China, with high efficiency in the degradation of petroleum hydrocarbons, phenolic compounds, and industrial pollutants (83, 84). We identified plasmid stabilization protein (ParE) and catabolically relevant genes for PET oligomer and monomer biodegradation and metabolism, including hydrolases, aryl-alcohol dehydrogenases, oxidoreductases, and redox enzymes (47). These similarities suggest that the integrated genomic island in strains 10/13.2 originated from an environmental *Pseudomonas* plasmid associated with aromatic compound metabolism, solvent tolerance, and xenobiotic transformation.

**Fig 5:**
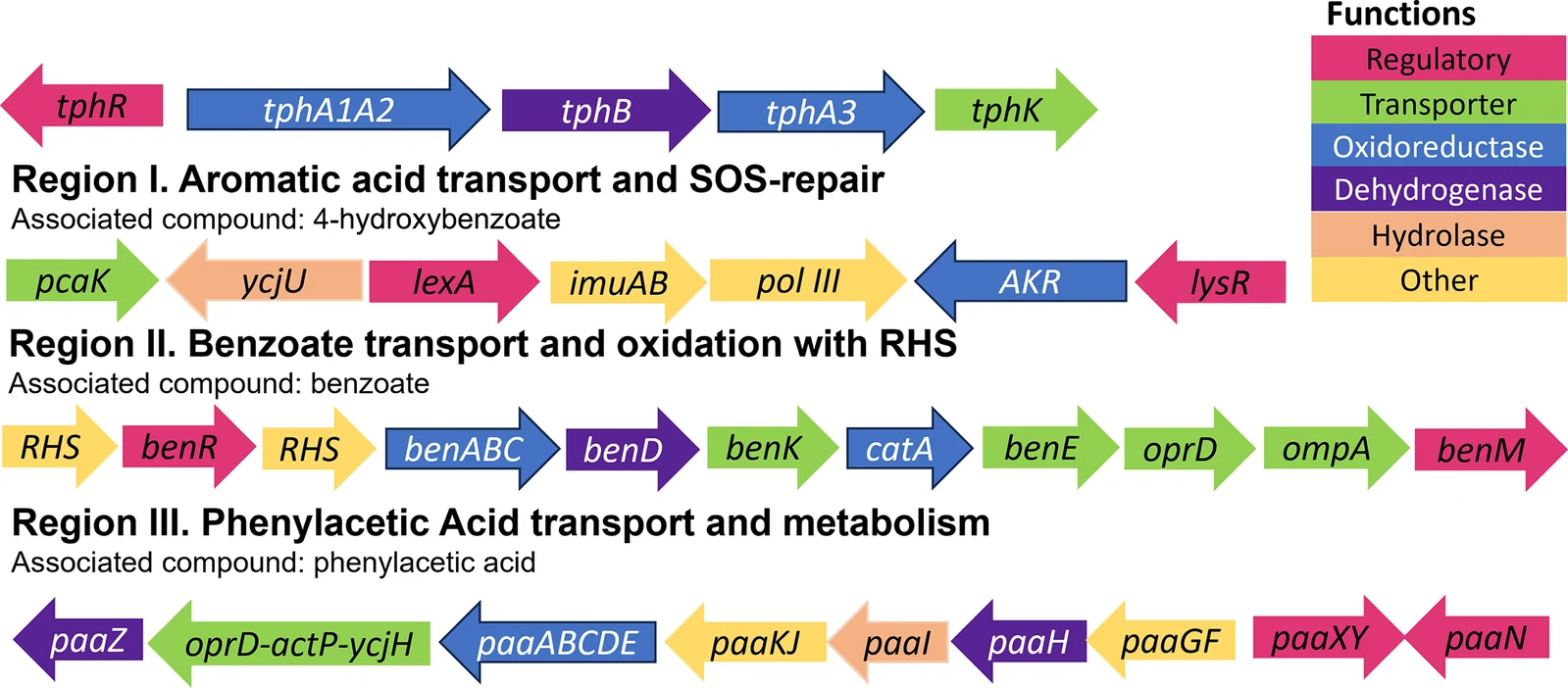
Accessory aromatic metabolism cassettes associated with genomic islands in *Pseudomonas* strain 10/13.2. Linear gene schematics compare a canonical terephthalate-associated metabolic cassette with accessory aromatic metabolism regions identified near predicted genomic islands. The canonical TPA cassette includes genes associated with terephthalate regulation and oxidation, including *tphR, tphA1A2, tphB, tphA3*, and *tphK* in *Pseudomonas umsongensis* BS3657. In contrast, the accessory regions in our bacterial consortium contain genes associated with aromatic acid uptake and stress-linked DNA repair, including a *pcaK*-like transporter (homologous to *tphK*), hydrolase, *lexA, imuAB*, an error-prone polymerase, reductase, and *lysR*. Additional cassettes include benzoate/catechol metabolism genes (*benABC, benD, benK, catA, benE,* and *benM*) adjacent to RHS (rearrangement hotspot) proteins and transport-associated genes (*oprD* and *ompA*), as well as a phenylacetic acid metabolism cassette containing *paaX, paaY, paaFGHIF, paaK, paaABCDE, paaZ, actP, oprD*, and *paaN*.

To evaluate whether this genomic island enables TPA metabolism, we compared canonical TPA gene cassettes seen in other *Pseudomonas* species with the aromatic degradation modules seen in this genomic region, shown in Fig. 5 (20, 85). A distinct TPA-specific *tph* cassette was not identified. However, the region contained multiple aromatic metabolism modules that could support transformation and assimilation of PET-adjacent aromatic intermediates. Shown in Fig. 5, these included stress-response genes (*lexA* and *imuA*), potentially enhancing tolerance to xenobiotic and solvent stress, and a duplicated or homologous *pcaK*-like transporter with nearby regulatory genes. We observed elements previously implicated in aromatic acid transport and TPA uptake (47, 62, 86), as well as a benzoate-associated cassette, with two rearrangement hotspot elements (RHS) flanking the benzoate regulatory element. The presence of RHS elements flanking the benzoate regulator suggests that this aromatic catabolic region is embedded within a rearrangement-prone genomic island (87), consistent with repeated HGT-mediated remodeling of ancestral benzoate metabolism. A complete phenylacetic acid degradation cassette is adjacent to this benzoate metabolism cassette, enabling parallel routes for metabolizing structurally related aromatic compounds: benzoate-like substrates can enter catechol/ring-cleavage metabolism, while phenylacetate-like substrates with side chains can enter CoA-dependent phenylacetate metabolism.

As the region also encoded diverse and numerous hydrolases, aryl-alcohol dehydrogenases, FAD-dependent oxidoreductases, LLM flavin-dependent oxidoreductases, aldo/keto reductases, alkene reductases, MFS transporters, ABC-type uptake systems, multidrug/MFS-family efflux transporters, LysR-family regulators, and stress-response genes (File S3), this locus likely functions as a broad xenobiotic-processing island that integrates aromatic substrate uptake, oxidative transformation, detoxification, and regulation rather than a narrowly specialized TPA degradation cassette. Several additional conserved but poorly characterized proteins (DUF1302 and DUF1329) are interspersed within the cassette, which have been implicated in fatty acid metabolism, potentially containing esterase-like domains. Individual genes show homology to components of phenylpropionate, flavonoid (e.g., naringenin), lignin-derived aromatic, and phthalate-associated pathways. However, the overall organization is not typical of aromatic degradation modules, normally having more genetic organization, like those seen in Fig. 5.

These data suggest the ability of broader aromatic metabolism, integrating transport, oxidation-reduction, hydrolysis, regulation, and xenobiotic stress tolerance together beyond TPA specificity. Although *Pseudomonas* strain 9.2 also contained the benzoate cassette (data not shown)**,** it lacked the flanking RHS elements and adjacent aromatic cassettes, including the phenylacetic acid cassette, as observed in strains 10/13.2, suggesting relatively reduced aromatic-catabolic potential. Rather than indicating a reduced role in PET-associated metabolism overall, this pattern may reflect functional partitioning, with 9.2 contributing more strongly to EG metabolism, redox balancing, and detoxification of soluble intermediates, while strains 10/13.2 retain expanded aromatic uptake and catabolic capacity.

### Proteomic enrichment patterns suggest functional specialization within consortium

**Fig 6:**
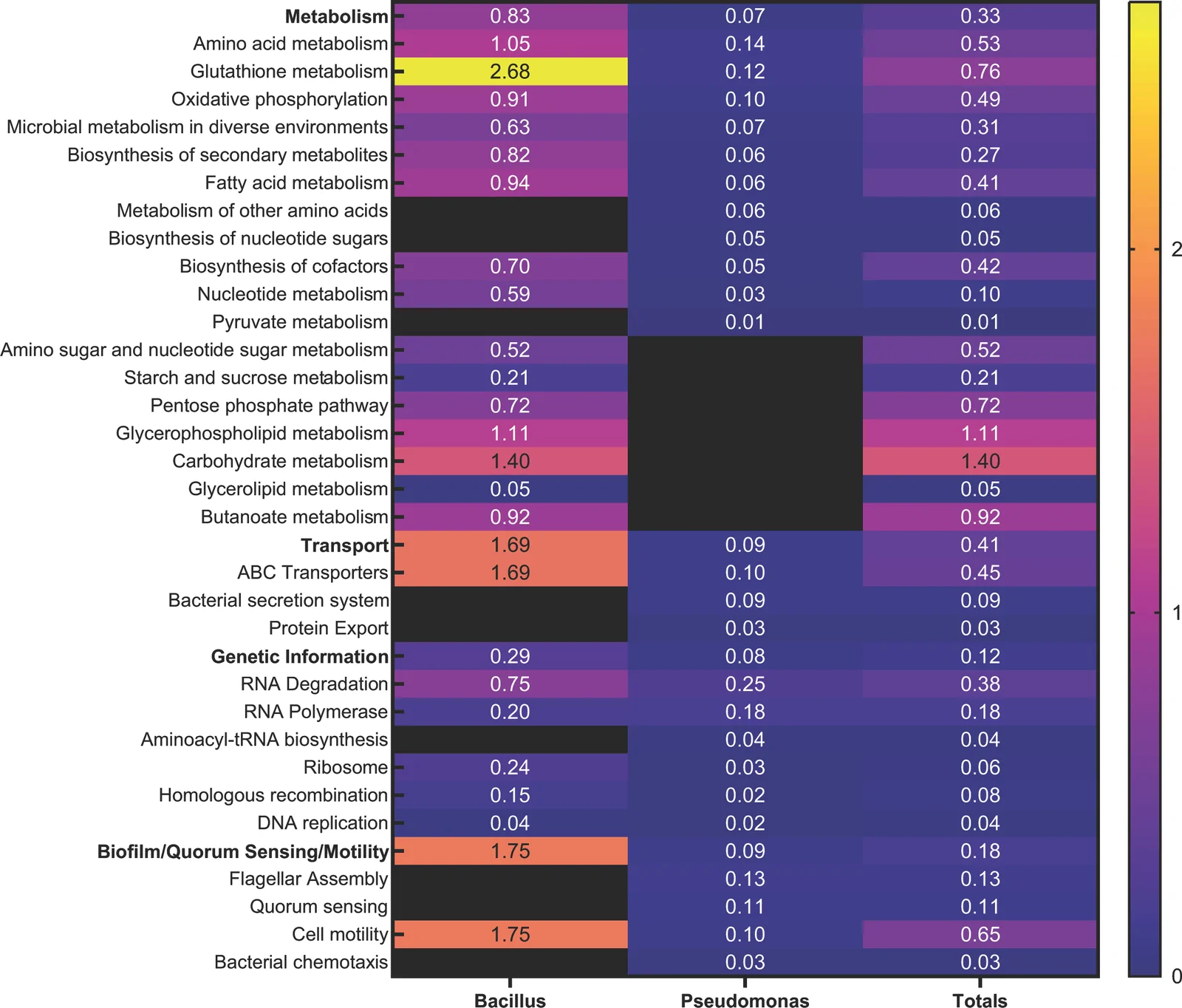
KEGG-mapped proteomic profiles reveal genus-level functional differences during PET-associated biofilm growth. Heatmap showing KEGG-assigned functional categories detected in the secreted/biofilm-associated proteome of *Bacillus* and *Pseudomonas* strains grown on PET. Values represent GroEL-normalized relative protein abundance for each functional category, with warmer colors indicating higher relative abundance and black cells indicating no detected proteins within that category. *Bacillus* showed higher normalized abundance, while *Pseudomonas* proteins were detected across a broader range of low-abundance metabolic, transport, secretion, and genetic information-processing categories. Total values summarize the combined contribution of both genera.

GroEL was used as a conserved housekeeping protein proxy for strain-level proteomic representation. Because monoculture proteomes under matched PET conditions were not generated, these data are interpreted as functional enrichment patterns within the consortium rather than direct evidence of induced expression. Following GroEL normalization (File S4), most proteins with defined KEGG assignments were associated with metabolism, representing approximately 78% of detected *Bacillus* proteins and 65% of detected *Pseudomonas* proteins. However, the abundance of these functions differed between genera, as seen in Fig. 6. *Bacillus* showed higher normalized abundance across all major functional categories, including metabolism, transport, oxidative phosphorylation, and biofilm/quorum sensing/motility. In contrast, *Pseudomonas* showed lower normalized abundance overall but broader representation across transport, secretion, redox, detoxification, and genetic information-processing categories, consistent with a more distributed role in soluble substrate uptake, stress tolerance, and intracellular metabolism.

*Bacillus* are well known as prolific protein secretors, biofilm formers, and biosynthetic specialists, particularly in the production of extracellular enzymes and secondary metabolites (88–90). Consistent with these roles, the *Bacillus* proteome was enriched in peptide uptake, amino acid catabolism, central carbon metabolism, oxidative phosphorylation, and nitrogen and carbon turnover (Table 3). High-abundance flagellin (FliC/Hag), detected only in *Bacillus 13.1*, further suggests a strain-specific role in motility, surface interaction, or biofilm remodeling during growth on PET.

**TABLE 3:** *Bacillus* proteome KEGG pathway mapping to metabolic function within the consortium

Functional category	Representative proteins	Metabolic interpretation
Peptide and amino acid scavenging	OppA/OppC/OppD/OppF, CarP/PepA, Ald, HutH, RocF/RocD, GabT, GlnA	Active uptake and metabolism of peptides and amino acids as carbon and nitrogen sources.
Central carbon metabolism	Eno, PfkA, Pyk, GapN, GpmI, Pgm, PdhA/PdhB, DlaT, Dld, AcaT/AtoB	Imported substrates are routed into glycolysis, pyruvate metabolism, acetyl-CoA production, and biosynthetic pathways.
TCA cycle and respiration	Mdh, IDH/Icd, SdhA, Ndh, CoxA/CoxB, QcrA, QoxA, ATPF1A/B/G/D, ATPF0B	Strong aerobic respiratory metabolism and ATP generation, consistent with active growth in the biofilm fraction.
Nitrogen and amino acid turnover	Ald, AmiF, HutH, RocF, RocD, Gabt, GlnA, DapE, Speb, Spee	Nitrogen recycling through alanine, histidine, arginine/ornithine, GABA/aminovalerate, and polyamine-associated metabolism.
Small carbon and membrane-derived substrate use	GlpQ/UgpQ, Pta, Ptb, NagA/NagE, CelB/ChbC, FabZ, LtaS	Use or recycling of glycerophosphoryl compounds, acetyl-phosphate/butyryl-phosphate intermediates, amino sugars, and membrane/cell-envelope components.
Redox balance and stress response	KatE, ALDH, MmsA/IolA, GLO1/GloA, DLD, Ndh, GroEL	Management of oxidative stress, aldehyde/semialdehyde intermediates, and protein-folding stress during surface-associated growth.
Aromatic-intermediate metabolism	HppD/HPD	Limited evidence for aromatic amino acid-derived intermediate metabolism; not sufficient to infer complete TPA/protocatechuate catabolism.
Motility and surface association	FliC/Hag, two-component system proteins	Motility, surface sensing, and environmental response during biofilm-associated growth.
Cell growth and maintenance	RpoC, ribosomal proteins, RecA, Ssb, Ndk, Pnp	Active transcription, translation, DNA repair, nucleotide metabolism, and protein maintenance.

In contrast, *Pseudomonas* proteomes contained a broader set of lower-abundance proteins associated with soluble substrate uptake, membrane export, redox balancing, alcohol and aldehyde metabolism, and intracellular assimilation (Table 4). Porins, ABC transporters, MFS transporters, TolC/RND-associated efflux proteins, and outer membrane proteins support active transport and trafficking of soluble PET intermediates, including the aromatic acid TPA (91, 92).

**TABLE 4:** *Pseudomonas* proteome KEGG pathway mapping to metabolic function within the consortium

Functional category	Proteins identified	Metabolic interpretation
Soluble substrate uptake	LivK/LivF/LivG, GltI/AatJ, AapJ/AapP, DppA, RbsB, GtsA/GlcE, GlpV, PstS, Sbp, PhnD	Broad import of amino acids, peptides, sugars, glycerol-like compounds, phosphate, sulfur compounds, and other soluble nutrients.
Peptide and amino acid utilization	CarP/PepA, PepN, Ggt, IlvC, LeuA/LeuB, GlnA, GDH2, HisB/HisD/HutU, ArgC/ArgH/OTC, RocD/RocF, TyrB	Active use of peptides and amino acids as carbon and nitrogen sources.
Central carbon assimilation	PfkA, FbaA, TpiA, GapA, Pgk, Eno, Pyk, Pgl, Gnd, TktA/TktB, TalA/TalB	Conversion of imported substrates through glycolysis, pentose phosphate metabolism, and central carbon pathways.
TCA cycle and acetyl-CoA metabolism	AceE, PDHA/PDHB, DLAT, DLD, OGDH/SucA, SucB, SucC/SucD, SdhA/SdhB/SdhC, Mdh, Icd, AcnB, Acs, Pta	Routing of soluble substrates into acetyl-CoA, TCA metabolism, and biosynthetic/energy-generating pathways.
Respiration and ATP generation	ATPF1A/B/G/D, ATPF0B, Ndh, CoxA/CoxB, CyoA/CyoB, CcoO/CcoP, QoxA, PetA/PetB/PetC	Aerobic respiratory metabolism and ATP production from imported carbon sources.
Redox balance and oxidative stress response	KatE, KatG, GSR/Gor, DLD, Ndh, Cytochrome Oxidases	Management of oxidative stress and redox cycling during biofilm-associated growth.
Alcohol and aldehyde metabolism	ExaA, AdhP, FrmA/AdhC, ALDH, AldB, MmsA/IolA, BetB/GbsA, GLO1/GloA	Detoxification or oxidation of alcohols, aldehydes, and semialdehydes, including potential processing of EG-derived or oxidative byproducts.
Aromatic-intermediate metabolism	HppD, HmgA, PhhA, TyrB, PheA, YdcJ, CsiD	Capacity for aromatic amino acid-derived acid metabolism and ring-cleavage-associated intermediate processing; not direct evidence of complete TPA catabolism.
Biofilm, motility, and surface response	FlgE, FliC/Hag, CheW, Mcp, Wza/GfcE, BapA	Surface sensing, motility, chemotaxis, biofilm matrix formation, and community-associated growth.
Membrane export and secretion	OprM, TolC, SecA/SecY/SecD, YidC, MsbA, MlaC/MlaD	Efflux, secretion, envelope maintenance, and tolerance to extracellular or toxic compounds.

**Fig 7:**
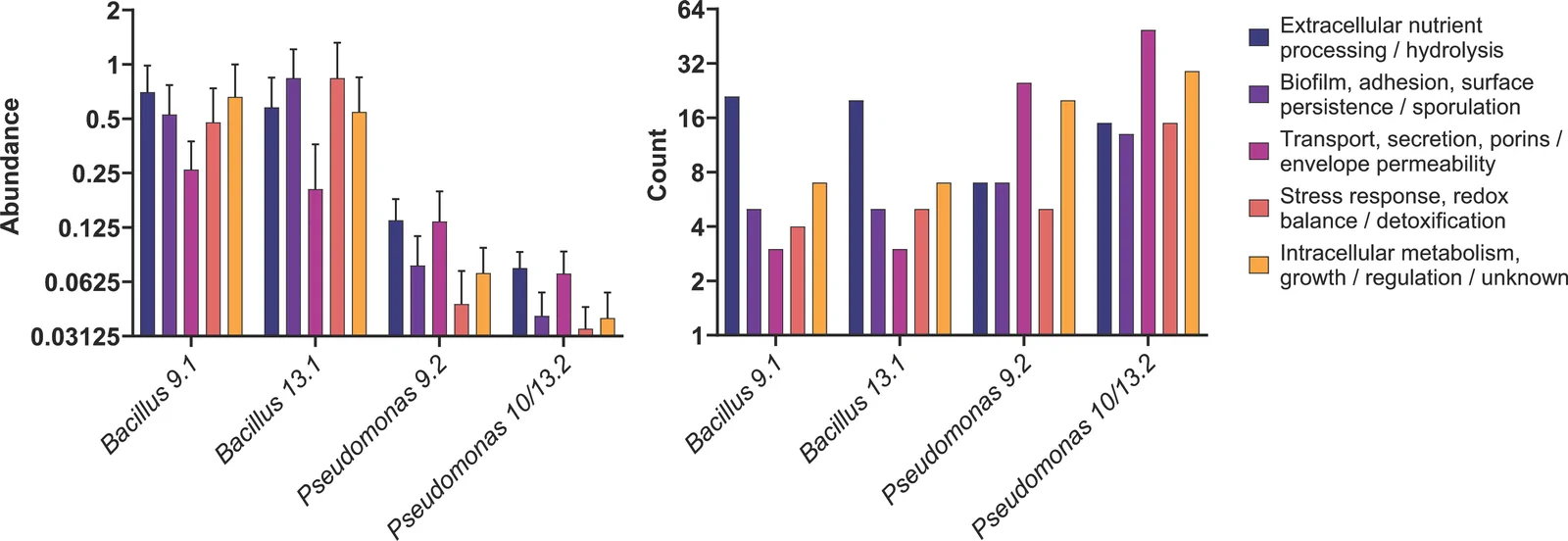
Non-KEGG proteome functional binning reveals genus-level differences in biofilm-associated protein abundance and diversity. Non-KEGG annotated proteins detected in the secreted/biofilm-associated proteome were manually assigned to five functional categories relevant to PET-associated growth: extracellular nutrient processing/hydrolysis, biofilm adhesion/surface persistence/sporulation, transport/secretion/porins/envelope permeability, stress response/redox balance/detoxification, and intracellular metabolism/growth/regulation/unknown functions. Protein counts indicate the number of proteins assigned to each category for each strain, while abundance values summarize the relative contribution of each functional category within the detected proteome. *Bacillus* strains showed stronger representation of extracellular nutrient processing, biofilm-associated, and persistence-related proteins, whereas *Pseudomonas* strains showed broader representation of transport, envelope permeability, redox/detoxification, and regulatory/metabolic functions.

To extend functional interpretation beyond KEGG-annotated proteins, proteins lacking defined KEGG pathway assignments were further sorted by PFAM/domain annotations into five categories relevant to PET-associated biofilm growth (File S5): extracellular nutrient processing/hydrolysis, biofilm adhesion/surface persistence/sporulation, transport/secretion/porins/envelope permeability, stress response/redox balance/detoxification, and intracellular metabolism/growth/regulation/unknown functions (Fig. 7). This PFAM-based analysis included 196 proteins that lacked KEGG pathway annotations within the secreted and biofilm-associated proteome and revealed clear genus-level differences in both protein abundance and functional diversity.

*Bacillus* strains were enriched in extracellular proteolysis/hydrolysis and biofilm-related proteins, consistent with roles identified in the KEGG analysis, further supporting *Bacillus* as a major contributor to extracellular enzyme production, possibly related to PET surface modification. In contrast, *Pseudomonas* strains displayed broader functional representation but lower relative abundance, mirroring the same pattern observed in KEGG-annotated proteins. Enriched *Pseudomonas* proteins included transporters, secretion-associated proteins, outer membrane proteins, redox and detoxification enzymes, and regulatory or metabolic proteins, supporting roles in soluble substrate uptake, envelope remodeling, stress tolerance, and metabolic flexibility.

Notably, CstA, which was identified above as an HGT-associated protein in *Bacillus*, was detected in *Pseudomonas* within the PET-associated proteome. Because CstA is associated with carbon starvation and nutrient-limited growth, its detection under PET biofilm conditions supports the functional relevance of starvation-response proteins during plastic-associated growth and illustrates shared strategies for persistence within the consortium. Expression of SAM-dependent methyltransferase and hydrolases, including previously identified EstP (35), is also present and is indicative of methyltransferase and hydrolysis activity, which may be directly involved in the hydrolysis of PET oligomers. Abundant alcohol and aldehyde dehydrogenases show active detoxification and metabolism of alcohols. This indicates that EG metabolism is only shown to be active in *Pseudomonas* strains. Overall, these proteomic data show that *Bacillus* are actively involved in the colonization and hydrolysis of the PET polymer and /BHET oligomer, while *Pseudomonas* play an important role in the breakdown, detoxification, and assimilation of oligomers (i.e., MHET) and monomers (TPA and EG).

### MHET modification expands PET oligomer assimilation pathways

Previously, ¹H NMR analysis of PET leachates following consortium incubation revealed unique methyl, glycol, acetaldehyde, and carboxylic acid resonances absent from abiotic controls, indicating operation of a specialized enzymatic cascade that couples PET oligomer hydrolysis with downstream modification to produce small, metabolizable aldehydes and organic acids. Interestingly, genome- and proteome-guided analyses did not identify a clear canonical MHETase homolog across consortium members, raising the possibility that MHET represents a potential metabolic bottleneck. Accordingly, we incubated individual isolates and the full consortium with MHET as the sole added substrate to assess whether MHET is directly hydrolyzed, transformed through alternative routes, or preferentially processed by specific community members.

**Fig 8:**
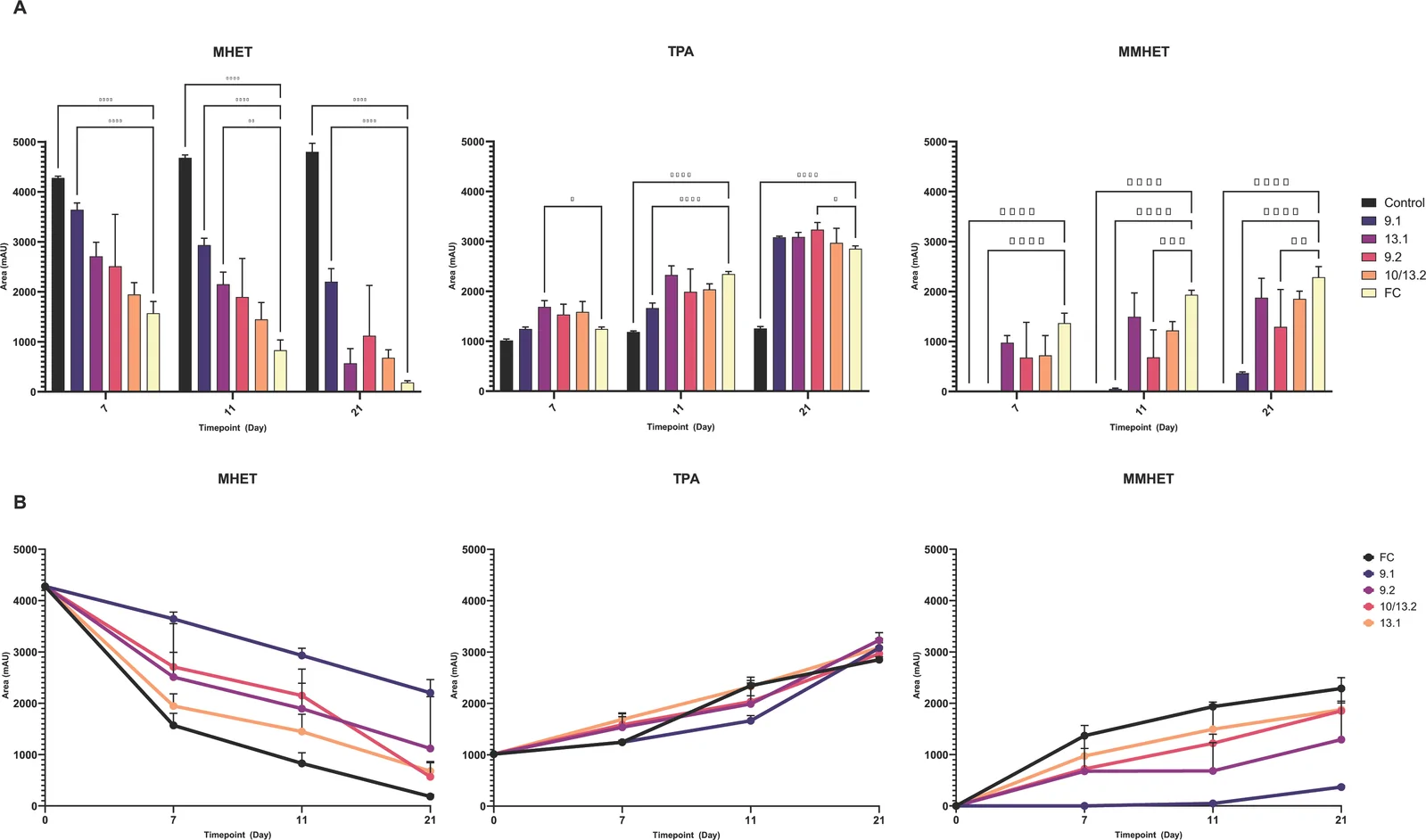
Temporal transformation dynamics of MHET, TPA, and MMHET across strains and the full consortium (FC). (**A**) Endpoint metabolite abundance at days 7, 11, and 21 following incubation with PET-derived substrates. MHET depletion, TPA accumulation, and MMHET production differed between strains over time. Statistical significance was determined independently for each metabolite using repeated-measures two-way ANOVA with Šídák’s multiple comparisons test comparing strains within each time point. Significant comparisons are indicated by asterisks (*P < 0.05, **P < 0.01, ***P < 0.001, ****P < 0.0001). (**B**) Longitudinal metabolite trajectories highlight differential substrate utilization and intermediate formation across taxa (normalized to Control). MHET generally declined over time while TPA and MMHET accumulated, though accumulation kinetics differed between isolates and near depletion after 21 days within the FC (shown in black). Data represent mean ± 1 SD from three biological replicates sampled longitudinally from the same flasks over 21 days. Control represents uninoculated media controls. FC = full consortium.

Strain-specific differences in MHET degradation and transformation were observed over the course of 21 days. When all consortium members were incubated together, the results showed greater overall MHET loss compared to the no-bacteria control and *Bacillus* strain 9.1 at all time points (Fig. 8). At day 11, the consortium showed greater MHET loss than *Bacillus* strain 13.1. The consortium was indistinguishable in regard to MHET degradation from the *Pseudomonas* strains. Thus, we concluded that within the consortium, the *Pseudomonas* strains play a pivotal role in MHET turnover. Accumulation of TPA was observed concurrently with MHET depletion, supporting hydrolytic conversion of MHET through the canonical PET degradation pathway. TPA increased over time in most biological treatments and was elevated relative to the abiotic control, confirming that MHET was actively converted to TPA. However, TPA accumulation alone did not fully account for the observed MHET loss, suggesting that MHET transformation may proceed through additional product pathways.

In addition to TPA, a secondary aromatic product was detected in biological treatments but was absent from abiotic controls (Fig. 8; Fig. S3 and S4). LC-MS analysis revealed an ESI feature at *m/z* 223.05, with retention time and fragmentation patterns consistent with a methylated MHET derivative (Table S3), tentatively assigned as MMHET. MMHET accumulated over time in several biological treatments, with the strongest signals observed in the full consortium and select isolates. Together, these results indicate that MHET turnover proceeds through both canonical hydrolysis to TPA and a secondary modification pathway involving the formation of the MMHET product. The strong MHET depletion and concurrent accumulation of both TPA and MMHET in the full consortium are consistent with previously observed transformation patterns during consortium incubation with PET and BHET (14, 35, 37). These findings support a model in which consortium metabolism enhances PET oligomer turnover and enables both hydrolytic conversion and secondary modification of aromatic intermediates.

These data suggest that when grown on the oligomer MHET, the strains methylate and hydrolyze MHET to reduce redox stress. MHET is not only an enzymatic inhibitor of PETase activity but also an amphipathic aromatic ester that causes significant membrane stress and redox pressure (93–95). These results are consistent with metabolic strategies commonly employed by HCB during aromatic compound transformation, including methylation, hydroxylation, and carboxylation reactions that facilitate detoxification and downstream metabolism of recalcitrant intermediates, particularly under biofilm-associated and oxygen-limited conditions (96). These findings show a modified enzymatic approach to PET degradation beyond hydrolysis, emphasizing methylation as a viable method for prolonged degradation.

### Conclusions

**Fig 9:**
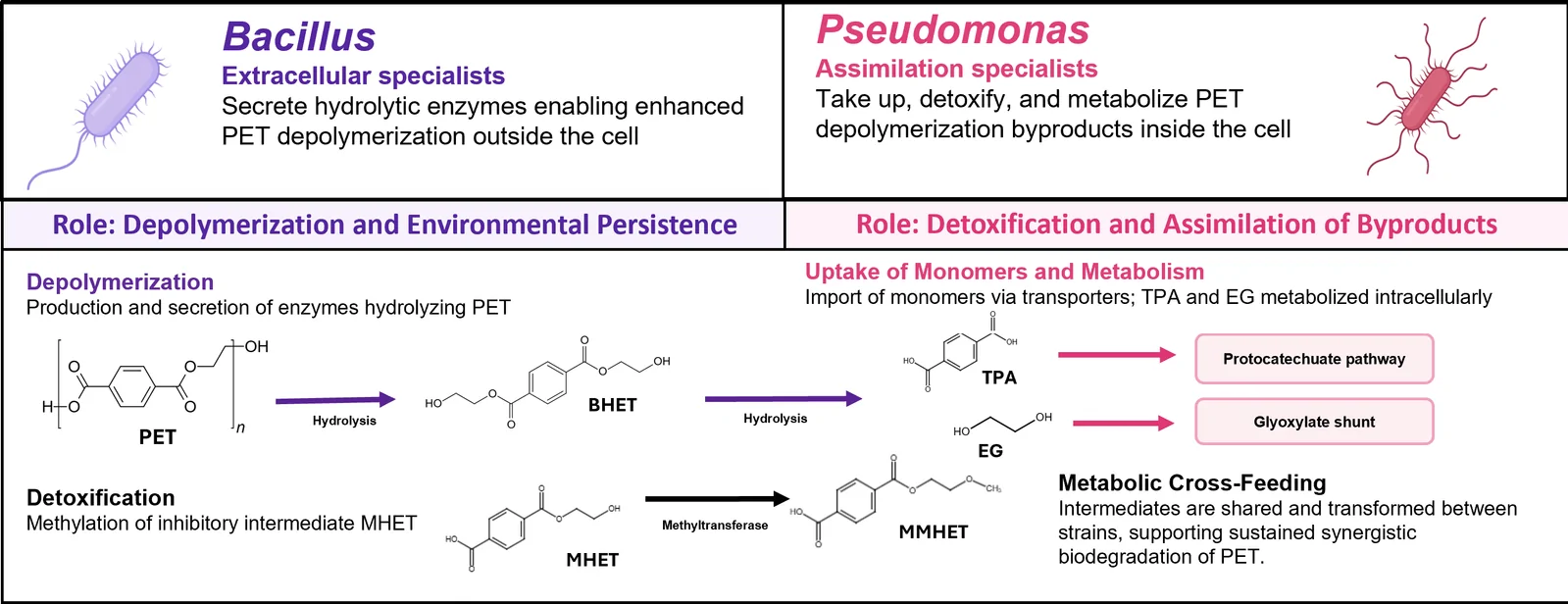
Proposed division of labor pathways among all consortium members for PET biodegradation. In the canonical hydrolytic pathway, PET is depolymerized into the soluble oligomers (2-hydroxyethyl) terephthalate and mono(2-hydroxyethyl) terephthalate, predominantly by *Bacillus* spp*.,* which are further hydrolyzed to TPA and EG. Monomers are assimilated by *Pseudomonas* strains. An alternative transformation pathway involves modified or methylated MHET derivatives (e.g., MMHET), which is employed by all strains.

Our findings support that PET biodegradation by the full consortium is not driven by a single strain or a single linear pathway but instead emerges from cooperative metabolism between community members (Fig. 9). Across chemical, genomic, and proteomic data sets, the full consortium showed the most complete PET-associated transformation, while individual isolates retained partial and strain-specific activities. This suggests that PET depolymerization, oligomer transformation, intermediate detoxification, and downstream aromatic metabolism are distributed across the community. *Bacillus* and *Pseudomonas* strains appear to have different functional strengths, with *Bacillus* contributing more strongly to extracellular hydrolysis, surface association, and persistence, while *Pseudomonas* are more strongly associated with aromatic transport, redox balancing, and downstream assimilation. However, these roles are not absolute. Rather, the consortium appears to rely on overlapping but unevenly distributed metabolic capacities that become most effective when the organisms are maintained together.

This interpretation is consistent with the original enrichment behavior of the consortium, in which *Bacillus* and *Pseudomonas* strains were recovered together from petroleum-polluted soils during selection for esterase activity (37). While this does not demonstrate obligate dependence, it suggests that these organisms were enriched under conditions where their activities may have been functionally linked. In this context, the full consortium is better understood as a metabolically integrated system rather than a collection of independent PET-degrading isolates.

This type of environmental PET biodegradation differs from simplified enzymatic PET hydrolysis, where PET biodegradation is often described through a PETase/MHETase-like activity (85). These systems have been essential for identifying enzymes capable of attacking PET and its oligomers, but they do not fully explain how PET is degraded in mixed environmental communities lacking these specific enzymes. In the full consortium described here, PET degradation requires more than ester bond cleavage. It also requires plastic surface colonization, soluble oligomer processing, aromatic intermediate transport, redox balancing, and tolerance of chemically stressful byproducts. Therefore, PET biodegradation in this system likely reflects the repurposing of ancestral hydrocarbon, aromatic, and xenobiotic metabolism of environmentally relevant compounds toward synthetic plastic substrates.

The genomic data suggest that predicted HGT events were primarily associated with general survival, biofilm formation, stress response, regulation, and core metabolic flexibility, rather than direct PET biodegradation. Although a small subset of HGT candidates were linked to PET-relevant functions, no specific transferred gene set was sufficient to explain PET degradation as a recently acquired pathway. Instead, these patterns suggest that *Bacillus* and *Pseudomonas* have likely shared ecological space over long periods, where traits supporting persistence, surface colonization, stress tolerance, and metabolic flexibility would be mutually beneficial. This interpretation is consistent with broader models in which horizontal gene transfer contributes to bacterial diversification and adaptation to new ecological niches (74). Thus, the HGT signals observed here are best understood as evidence of shared ecological adaptation and cooperative survival, rather than direct transfer of PET biodegradation capacity.

A more specific example of this metabolic flexibility is the xenobiotic-associated genomic island identified in *Pseudomonas* strains 10/13.2. This region contains genes associated with aromatic compound transport, benzoate metabolism, phenylacetic acid catabolism, stress response, and plasmid maintenance. While this island does not represent a complete PET degradation pathway, its size and composition suggest that it may have been acquired to expand upon the native ability of strains 10/13.2 to respond to aromatic and chemically stressful compounds, such as those generated during PET degradation. Importantly, PET-associated metabolism was not restricted to only these two *Pseudomonas* strains, as *Pseudomonas* strain 9.2 also showed evidence of monomer transformation. Thus, the genomic island is best interpreted as an accessory flexibility module that may enhance aromatic intermediate processing and xenobiotic stress tolerance within the consortium.

Proteomic data further support functional specialization across the consortium, although these roles were not exclusive. *Bacillus* strains showed stronger enrichment of secreted hydrolases, oxidative enzymes, and stress-associated proteins, consistent with a major role in extracellular processing of polymer-derived material and persistence under chemically stressful conditions. In contrast, *Pseudomonas* strains showed greater representation of transporters, oxidoreductases, aldehyde dehydrogenases, and surface-attachment proteins, suggesting a stronger role in biofilm formation, uptake of soluble intermediates, aromatic assimilation, and redox management. Together, these patterns support a metabolic handoff in which enzyme-rich extracellular depolymerization generates soluble intermediates that are then transported, modified, detoxified, or assimilated by other members of the community.

The MHET degradation data illustrate why this cooperation may be necessary. MHET is not only a central PET oligomer intermediate but also a bottleneck when downstream processing is incomplete. The stronger MHET depletion observed in the full consortium suggests that continued oligomer turnover requires coordinated hydrolysis, transport, and intermediate management rather than hydrolysis alone. Although TPA accumulation supports the canonical conversion of MHET to terephthalic acid, MHET loss was not fully explained by TPA formation. The detection of a secondary aromatic product in biological samples, but not abiotic controls, suggests that MHET may also enter a biologically associated modification route. LC-MS analysis identified an ESI feature at 223.05 *m/z*, with retention time and fragmentation patterns consistent with a methylated MHET derivative, tentatively assigned as methyl mono-(2-hydroxyethyl) terephthalate, or MMHET. We propose that MMHET formation may help manage PET-derived intermediates by altering MHET reactivity, solubility, transport, or toxicity, although its specific biological role remains to be tested directly. Together, these findings suggest that this consortium degrades PET by repurposing existing hydrocarbon, aromatic, and stress-response metabolism, rather than by acquiring a dedicated PET degradation pathway. The consortium repurposes ester-cleaving, aromatic metabolism, transport, stress-response, and redox-balancing functions toward PET-derived substrates. While individual strains can degrade oligomers in various degrees of efficiency, sustained PET turnover requires these functions to operate together, allowing the community to depolymerize PET, process soluble oligomers, and manage chemically stressful intermediates such as MHET. In this context, MMHET formation may reflect an auxiliary route for intermediate management rather than a separate endpoint of degradation. In this system, plastic biodegradation is therefore not the product of a single novel pathway but a multi-step, cooperative process built from ancestral metabolic tools acting together in a new ecological context.

## Supplementary Material

Reviewer comments

## Data Availability

The data are contained within the article.

## SUPPLEMENTAL MATERIAL

File S1 msystems.00829-26-s0001.xlsx DarkHorse HGT candidates.

File S2 msystems.00829-26-s0002.xlsx HGT candidates: KEGG and DarkHorse taxonomic classifications.

File S3 msystems.00829-26-s0003.xlsx Predicted genomic island in *Pseudomonas* sp. 10.

File S4 msystems.00829-26-s0004.xlsx Proteomic functional annotations.

File S5 msystems.00829-26-s0005.xlsx PFAM categorical proteomic assignment.

Supplemental Figures and Tables msystems.00829-26-s0006.docx Figures S1-S4 and Tables S1-S3.

OPEN PEER REVIEW reviewer-comments.pdf An accounting of the reviewer comments and feedback.

ASM does not own the copyrights to Supplemental Material that may be linked to, or accessed through, an article. The authors have granted ASM a non-exclusive, world-wide license to publish the Supplemental Material files. Please contact the corresponding author directly for reuse.
