# Supplementary material for: Ancestral hydrocarbon metabolism enables PET degradation by a natural bacterial consortium
Source: mSystems. 2026 Aug 21;11(9):e00829-26. doi: 10.1128/msystems.00829-26 (PMC13595931; doi:10.1128/msystems.00829-26)
Supplement: Supplementary file 1 [file reviewer-comments.pdf]

ASM | mSystems

Ancestral Hydrocarbon Metabolism Enables PET Degradation by a Natural Bacterial Consortium

Sabrina Edwards, Danny Rice, Patricio Palomino, Irene Newton, and Jay Mellies

Corresponding Author(s): Jay Mellies, Reed College

Review Timeline:

Submission Date:	June 29, 2026
Editorial Decision:	July 10, 2026
Revision Received:	July 23, 2026
Accepted:	July 29, 2026

Editor: Ashley Shade

Reviewer(s): The reviewers have opted to remain anonymous.

Transaction Report:

(Note: With the exception of the correction of typographical or spelling errors that could be a source of ambiguity, letters and reports are not edited. The original formatting of letters and referee reports may not be reflected in this compilation.)

DOI: https://doi.org/10.1128/msystems.00829-26

Re: mSystems00829-26 (**Ancestral Hydrocarbon Metabolism Enables PET Degradation by a Natural Bacterial Consortium**)

Dear Prof. Jay Mellies:

Thank you for the privilege of reviewing your work. Below you will find my comments, instructions from the mSystems editorial office, and the reviewer comments.

Dear Authors - Thank you for your careful revisions. As you will read, the original reviewer was overall quite satisfied with the revisions and found the paper to be interesting and timely. However, the reviewer still suggests that some re-calibration of the language around the claims of synergistic conversion and division of labor is needed for the results to be more precisely aligned with the data presented, and I generally agree. Thus, I consider these to be minor, text-only revisions to temper wording and to present these possibilities as hypotheses where appropriate. I very much look forward receiving your revision. -Ashley

Please return the manuscript within 60 days; if you cannot complete the modification within this time period, please contact me. If you do not wish to modify the manuscript and prefer to submit it to another journal, notify me immediately so that the manuscript may be formally withdrawn from consideration by mSystems.

Revision Guidelines
To submit your modified manuscript, log into the submission site at https://msystems.msubmit.net/cgi-bin/main.plex. Go to Author Tasks and click the appropriate manuscript title to begin. The information you entered when you first submitted the paper will be displayed; update this as necessary. Note the following requirements:

• Upload point-by-point responses to the issues raised by the reviewers in a file named "Response to Reviewers," NOT in your cover letter.
• Upload a compare copy of the manuscript (without figures) as a "Marked-Up Manuscript" file.
• Upload a clean .DOC/.DOCX version of the revised manuscript and remove the previous version.
• Each figure must be uploaded as a separate, editable, high-resolution file (TIFF or EPS preferred), and any multipanel figures must be assembled into one file.
• Any supplemental material intended for posting by ASM should be uploaded with their legends separate from the main manuscript. You can combine all supplemental material into one file (preferred) or split it into a maximum of 10 files with all associated legends included.

For complete guidelines on revision requirements, see our Submission and Review Process webpage. Submission of a paper that does not conform to guidelines may delay acceptance of your manuscript.

Data availability: ASM policy requires that data be available to the public upon online posting of the article, so please verify all links to sequence records, if present, and make sure that each number retrieves the full record of the data. If a new accession number is not linked or a link is broken, provide mSystems production staff with the correct URL for the record. If the accession numbers for new data are not publicly accessible before the expected online posting of the article, publication may be delayed; please contact production staff (mSystems@asmusa.org) immediately with the expected release date.

Publication Fees: For information on publication fees and which article types are subject to charges, visit our website. If your manuscript is accepted for publication and any fees apply, you will be contacted separately about payment during the production process; please follow the instructions in that e-mail. Arrangements for payment must be made before your article is published.

ASM Membership: Corresponding authors may join or renew ASM membership to obtain discounts on publication fees. Need to upgrade your membership level? Please contact Customer Service at Service@asmusa.org.

The ASM Journals program strives for constant improvement in our submission and publication process. Please tell us how we can improve your experience by taking this quick Author Survey.

Thank you for submitting your paper to mSystems.

Sincerely,
Ashley Shade
Editor
mSystems

Reviewer #2 (Comments for the Author):

I thank the authors for a substantially revised manuscript. Several earlier concerns are well addressed: the ecological terminology has been appropriately tightened, statistical analysis has been added, and the proteomic interpretation now states the limitation regarding biomass and the lack of monoculture controls.
My remaining concern is singular: The two central claims i.e. synergistic/community-level MHET conversion and a Bacillus/Pseudomonas division of labor, are not supported by the figures. For the MHET assay they are contradicted by the authors' own statistics. My specific points are as follows.
1. Figure 8 shows a quantitative difference in MHET turnover, not a consortium-exclusive effect. The full consortium sits at the efficient end of the range, but the two Pseudomonas isolates (9.2 and 10/13.2) fall within the plotted error of it; only the Bacillus strains (9.1, 13.1) and the no-cell control separate clearly. This is a difference in degree, not the qualitative, all-or-nothing effect the manuscript's framing conveys. The authors describe the system as exhibiting "synergistic PET degradation mediated through a cooperative division of labor, as complete PET mineralization occurs when all strains are present" (89 - 91) and as a "community-level" process (30, 498), and they offer the MHET result as evidence that "continued oligomer turnover requires coordinated hydrolysis, transport, and intermediate management rather than hydrolysis alone" (480 - 481). Figure 8 does not support that reading. The consortium is more efficient than Bacillus and the control, but the panel shows the community performing the same MHET conversion as the individual Pseudomonas isolates already perform. A consortium that matches its best-performing members is not evidence of a synergistic or community-level function.
2. The statistics argue against the synergy claim. The significant contrasts are FC vs Control (all timepoints), FC vs Bacillus 9.1 (all timepoints), and FC vs Bacillus 13.1 (day 11 only). There is no significant difference between the full consortium and either Pseudomonas isolate (9.2 or 10/13.2) at any timepoint. Synergy or emergence requires the consortium to exceed its best-performing member (here the Pseudomonas strains) so these statistics cannot support a community-level claim. They show the consortium matching a single Pseudomonas strain while out-performing only the Bacillus isolates and the no-cell control. "Synergistic," and "community-level" should be removed from the MHET results, and the finding restated as: the consortium is statistically indistinguishable from the individual Pseudomonas isolates.
3. MMHET formation is also not consortium-exclusive. Figure 8A shows MMHET produced individually by Pseudomonas 9.2 and 10/13.2 and by Bacillus 13.1. Since both MHET depletion and MMHET formation are reproduced by single strains, the MHET assay cannot serve as evidence for a division of labor and should not be cited as such. A point the authors themselves concede in the Figure 9 legend, where the methylated-MHET pathway is described as "employed by all strains" (869).

I thank the authors for a substantially revised manuscript. Several earlier concerns are well addressed: the ecological terminology has been appropriately tightened, statistical analysis has been added, and the proteomic interpretation now states the limitation regarding biomass and the lack of monoculture controls.

My remaining concern is singular: The two central claims i.e. synergistic/community-level MHET conversion and a Bacillus/Pseudomonas division of labor, are not supported by the figures. For the MHET assay they are contradicted by the authors' own statistics. My specific points are as follows.

1. Figure 8 shows a quantitative difference in MHET turnover, not a consortium-exclusive effect. The full consortium sits at the efficient end of the range, but the two Pseudomonas isolates (9.2 and 10/13.2) fall within the plotted error of it; only the Bacillus strains (9.1, 13.1) and the no-cell control separate clearly. This is a difference in degree, not the qualitative, all-or-nothing effect the manuscript's framing conveys. The authors describe the system as exhibiting "synergistic PET degradation mediated through a cooperative division of labor, as complete PET mineralization occurs when all strains are present" (89 - 91) and as a "community-level" process (30, 498), and they offer the MHET result as evidence that "continued oligomer turnover requires coordinated hydrolysis, transport, and intermediate management rather than hydrolysis alone" (480 - 481). Figure 8 does not support that reading. The consortium is more efficient than Bacillus and the control, but the panel shows the community performing the same MHET conversion as the individual Pseudomonas isolates already perform. A consortium that matches its best-performing members is not evidence of a synergistic or community-level function.

The authors agree with the reviewer's comments that the quantitative difference in MHET turnover is not a consortium effect. The section describing the MHET degradation has been edited as suggested (see response to comment 2). In addition, beginning on line 89 the text reads, "This *Bacillus-Pseudomonas* consortium degrades PET through a cooperative division of labor." Beginning on line 593, "In this system, plastic biodegradation is therefore not the product of a single novel pathway but a multi-step, cooperative process built from ancestral metabolic tools acting together in a new ecological context."

2. The statistics argue against the synergy claim. The significant contrasts are FC vs Control (all timepoints), FC vs Bacillus 9.1 (all timepoints), and FC vs Bacillus 13.1 (day 11 only). There is no significant difference between the full consortium and either Pseudomonas isolate (9.2 or 10/13.2) at any timepoint. Synergy or emergence requires the consortium to exceed its best-performing member (here the Pseudomonas strains) so these statistics cannot support a community-level claim. They show the consortium matching a single Pseudomonas strain while out-performing only the Bacillus isolates and the no-cell control. "Synergistic," and "community-level" should be removed from the MHET results, and the finding restated as: the consortium is statistically indistinguishable from the individual Pseudomonas isolates.

As suggested, the manuscript has been edited, beginning on line 437, “. When all consortium members were incubated together, results showed greater overall MHET loss compared to the no-bacteria control and *Bacillus* strain 9.1 at all time points (Figure 8). At day 11, the consortium showed greater MHET loss than *Bacillus* strain 13.1. The consortium was indistinguishable in regard to MHET degradation from the *Pseudomonas* strains. Thus, we concluded that within the consortium, the *Pseudomonas* strains play a pivotal role in MHET turnover.”

3. MMHET formation is also not consortium-exclusive. Figure 8A shows MMHET produced individually by Pseudomonas 9.2 and 10/13.2 and by Bacillus 13.1. Since both MHET depletion and MMHET formation are reproduced by single strains, the MHET assay cannot serve as evidence for a division of labor and should not be cited as such. A point the authors themselves concede in the Figure 9 legend, where the methylated-MHET pathway is described as "employed by all strains" (869).

Thank you for the comment. The authors agree that the MHET assay and MMHET formation in Figure 8 do not indicate a division of labor. See comment 2 for the edited text in the MHET section. The division of labor is clearly indicated by other data, particularly those illustrated in Figure 7.

Re: mSystems00829-26R1 (**Ancestral Hydrocarbon Metabolism Enables PET Degradation by a Natural Bacterial Consortium**)

Dear Prof. Jay Mellies:

Your manuscript has been accepted, and I am forwarding it to the ASM production staff for publication. Your paper will first be checked to make sure all elements meet the technical requirements. ASM staff will contact you if anything needs to be revised before copyediting and production can begin. Otherwise, you will be notified when your proofs are ready to be viewed.

Data Availability: ASM policy requires that data be available to the public upon online posting of the article, so please verify all links to sequence records, if present, and make sure that each number retrieves the full record of the data. If a new accession number is not linked or a link is broken, provide production staff with the correct URL for the record. If the accession numbers for new data are not publicly accessible before the expected online posting of the article, publication may be delayed; please contact ASM production staff immediately with the expected release date.

Publication Fees: For information on publication fees and which article types have charges, please visit our website. We have partnered with Copyright Clearance Center (CCC) to collect author charges. If fees apply to your paper, you will receive a message from no-reply@copyright.com with further instructions. For questions related to paying charges through RightsLink, please contact CCC at ASM_Support@copyright.com or toll free at +1-877-622-5543. CCC makes every attempt to respond to all emails within 24 hours.

ASM Membership: Corresponding authors may join or renew ASM membership to obtain discounts on publication fees. Need to upgrade your membership level? Please contact Customer Service at Service@asmusa.org.

Embargo Policy: A press release may be issued as soon as the manuscript is posted on the mSystems Latest Articles webpage. The corresponding author will receive an email with the subject line "ASM Journals Author Services Notification" when the article is available online.

Cover Image Submissions: If you would like to submit a potential Cover Image, please email a file and a short legend to msystems@asmusa.org. Please note that we can only consider images that (i) the authors created or own and (ii) have not been previously published. By submitting, you agree that the image can be used under the same terms as the published article. Image File requirements: TIF/EPS, 7.5 inches wide by 8.25 inches tall (at least 2,250 pixels wide by 2,475 pixels tall), minimum 300 dpi resolution (600 dpi preferred), RGB, and no figure elements, e.g., arrows or panel labels. The legend should be a short description of the image, 1-2 sentences recommended. Please download and use this interactive template in Adobe the https://journals.asm.org/pb-assets/html/asm-cover-sizing-tool.html to ensure that your proposed cover image meets our size requirements (https://journals.asm.org/pb-assets/pdf-text-excel-files/ASM-Interactive-Sizing-Cover-Template-1715689791.pdf).

Thank you for submitting your paper to mSystems.

Sincerely,
Ashley Shade
Editor
mSystems
