# Supplementary material for: Ancestral hydrocarbon metabolism enables PET degradation by a natural bacterial consortium
Source: mSystems. 2026 Aug 21;11(9):e00829-26. doi: 10.1128/msystems.00829-26 (PMC13595931; doi:10.1128/msystems.00829-26)
Supplement: Supplementary file 2 [file msystems.00829-26-s0006.docx]

**Figure S1: Average Distribution of DarkHorse lineage probability index (LPI) scores across consortium member genomes.** For each consortium member, all predicted genes were assigned a lineage probability index (LPI) score using DarkHorse to assess potential horizontal gene transfer. Predicted HGT candidates were grouped by kingdom-level taxonomic assignment and compared across consortium members. LPI scores <0.20 coincided with non-bacterial assignments, including Eukaryota, Fungi, and Viruses, were excluded from downstream bacterial HGT analyses to minimize false-positive donor assignments and focus interpretation on bacterial gene exchange. After filtering, bacterial LPI assignments were retained for taxonomic and functional analysis of putative horizontally acquired genes.

***Table S1: Distribution of DarkHorse consortium genes and their lineage probability index (LPI) score***

| **Bacillus** | | | | | | **Pseudomonas** | | | | | |
| --- | --- | --- | --- | --- | --- | --- | --- | --- | --- | --- | --- |
| **9.1 HGT Breakdown** | | | **13.1 HGT Breakdown** | | | **9.2 HGT Breakdown** | | | **10/13.2 HGT Breakdown** | | |
|  | *(n)* | *LPI* |  | *(n)* | *LPI* |  | *(n)* | *LPI* |  | *(n)* | *LPI* |
| **Viruses** | **14** | **0.003** | **Viruses** | **115** | **0.016** | **Viruses** | **11** | **0.002** | **Viruses** | **6** | **0.002** |
| *Heunggongvirae* | 14 | 0.003 | *Heunggongvirae* | 115 | 0.016 | *Heunggongvirae* | 11 | 0.002 | *Heunggongvirae* | 6 | 0.002 |
| **Eukaryota** | **2** | **0.105** |  |  |  | **Eukaryota** | **32** | **0.004** | **Eukaryota** | **11** | **0.022** |
| *Metazoa* | 2 | 0.105 |  |  |  | *Fungi* | 2 | 0.004 | *Fungi* | 1 | 0.022 |
|  |  |  |  |  |  | *Metazoa* | 3 | 0.006 | *Metazoa* | **8** | **0.118** |
|  |  |  |  |  |  |  |  |  | *Viridiplantae* | **3** | **0.024** |
| **Bacteria** | **5008** | **0.407** | **Bacteria** | **4756** | **0.398** | **Bacteria** | **5388** | **0.418** | **Bacteria** | **4528** | **0.422** |
|  |  |  | *Thermodesulfobacteriota* | 1 | 0.421 | *Cyanobacteriota* | 2 | 0.418 | *Nitrospirota* | 2 | 0.455 |
|  |  |  | *Spirochaetota* | 1 | 0.421 | *Campylobacterota* | 2 | 0.418 | *Cyanobacteriota* | 2 | 0.424 |
|  |  |  | *Cyanobacteriota* | 2 | 0.398 | *Mycoplasmatota* | 2 | 0.418 | *Bacteroidota* | 26 | 0.422 |
|  |  |  | *Myxococcota* | 2 | 0.398 | *Bacteroidota* | 7 | 0.418 | *Actinomycetota* | 75 | 0.426 |
| *Bacteroidota* | 2 | 0.42 | *Bacteroidota* | 3 | 0.421 | *Actinomycetota* | 18 | 0.419 | *Bacillota* | 149 | 0.427 |
| *Actinomycetota* | 3 | 0.42 | *Actinomycetota* | 7 | 0.421 | *Bacillota* | 15 | 0.419 | *Pseudomonadota* | 4274 | 0.591 |
| *Campylobacterota* | 6 | 0.42 | *Campylobacterota* | 10 | 0.421 | *Pseudomonadota* | 5342 | 0.593 |  |  |  |
| *Pseudomonadota* | 260 | 0.407 | *Pseudomonadota* | 258 | 0.408 |  |  |  |  |  |  |
| *Bacillota* | 4737 | 0.617 | *Bacillota* | 4472 | 0.618 |  |  |  |  |  |  |

DarkHorse LPI scores were calculated for all genes in each consortium genome to identify putative horizontally transferred genes. Lower LPI values indicate reduced lineage consistency and greater likelihood of horizontal acquisition. Genes with LPI ≤ 0.50 were considered HGT candidates; genes with LPI < 0.20 were excluded from this analysis to focus on bacterial candidate transfers.

**Figure S2: Unfiltered class- and family-level taxonomic distribution of predicted HGT candidates across consortium members.**
Predicted HGT candidates were assigned to taxonomic class and family based on lowest LPI scores and plotted as gene counts for each consortium member. This pre-filtered dataset includes all candidate assignments, including rare taxa represented by fewer than three genes. The figure illustrates the initial taxonomic breadth of putative HGT signals prior to removal of low-count or non-bacterial assignments used in downstream analyses. Distributions are shown separately for Bacillus strains 9.1 and 13.1, Pseudomonas sp. 9.2, and the combined Pseudomonas sp. 10/13.2 group.

| **Table S2: Predicted horizontally transferred genes associated with PET-relevant^±^ supportive functions in the consortium.** | | | | | |
| --- | --- | --- | --- | --- | --- |
| **DarkHorse Class** | **DarkHorse Genus** | **Consortium Genus** | **General Function** | **Predicted Gene Annotation** | **KEGG Classification** |
| Actinomycetes | Arthrobacter | Pseudomonas | Hydrolase | 3-oxoadipate enol-lactonase (PcaD) | γ-proteobacteria, Pseudomonas |
|  |  |  | Oxidoreductase | α-Ketoglutaric semialdehyde dehydrogenase |  |
|  |  |  | Oxidoreductase | NirD (ferredoxin-like protein) |  |
|  |  |  | Regulator | HTH/LysR-family transcriptional regulator |  |
|  |  |  | Regulator | LysR-family transcriptional regulator |  |
|  |  |  | Transporter | PBP2_ToxR-like periplasmic binding protein |  |
|  | Corynebacterium |  | Regulator | LysR-family transcriptional regulator |  |
|  | Mycobacterium | Bacillus | Oxidoreductase | Cytochrome P450 (CYP) |  |
| Bacilli | Enterococcus | Pseudomonas | Regulator | SutA Transcriptional Regulator |  |
|  | Priestia |  | Glycosylation | GT-A fold glycosyltransferase |  |
|  |  |  | Hydrolase | MenH (α/β hydrolase) |  |
|  |  |  | Oxidoreductase | Phthalate dioxygenase reductase-like |  |
|  |  |  | Oxidoreductase | Aromatic ring-hydroxylating dioxygenase |  |
|  |  |  | Oxidoreductase | Phthalate dioxygenase reductase-like |  |
|  |  |  | Oxidoreductase | SDR family oxidoreductase |  |
|  |  |  | Regulator | LysR-family transcriptional regulator |  |
|  |  |  | Transporter | TRAP transporter solute receptor (TAXI) |  |
|  |  |  | Transporter | TRAP-type fused permease (COG4666) |  |
|  |  |  | Transporter | Type 2 periplasmic binding protein |  |
| γ-proteobacteria | Escherichia | Bacillus | Oxidoreductase | NADH dehydrogenase (Ndh) | Bacilli, Bacillus |
|  | Klebsiella |  | Glycosylation | WcaA (capsule glycosyltransferase) |  |
|  |  |  | Glycosylation | PimA-like mannosyltransferase (GT4) |  |
|  |  |  | Hydrolase | MenH_SHCHC synthase |  |
|  |  |  | Oxidoreductase | Epoxyqueuosine reductase (QueG) |  |
|  |  |  | Regulator | CstA Carbon Starvation Protein |  |
| Chitinophagaceae | Chitinophagia | Pseudomonas | Transporter | CrgA-like periplasmic binding protein (PBP2) | β-proteobacteria, Paraburkholderia |
| *^±^* PET-relevant functions were defined as genes with potential roles in polymer hydrolysis or biotransformation, aromatic and alcohol metabolism, transport, regulation, redox activity, and stress-associated adaptation. Functional categories include hydrolases, oxidoreductases, regulators, glycosylation-associated enzymes, and transporters. | | | | | |

**Figure S3. HPLC chromatograms of consortium incubated with MHET overlaid with combined standards containing TPA, MHET and BHET show ‘Mystery’ Compound.** Retention times (in minutes) for aromatic monomers TPA and the oligomers MHET and BHET were found to be at 0.52, 0.68 and 0.85, respectively. A compound observed when incubated with MHET solely, shows a ‘mystery’ compound at 0.77 minutes, distinct from any canonical PETase byproducts.

**Figure S4.** **HPLC chromatogram overlay of all strains compared with abiotic control (CON) and the full consortium (FC).** The abiotic control (CON) shown in dark orange shows a small amount of autohydrolysis of MHET to TPA. *Bacillus* strains, 9.1 in purple and 13.1 in blue. *Pseudomonas* strains are shown in light orange (9.2), black (10), and green (13.2). All five strains combined are shown in burgundy (FC). The presence of the mystery compound peaks in the FC and does not appear to be present when *Bacillus* strain 9.1 is incubated with MHET. Varying hydrolysis and conversion rates between strains indicates individual strain handling of MHET differs.

**Table S3: LC–MS (ESI–) Summary of PET-Related Intermediates**

| **Compound** | **Molecular Formula** | **Neutral Exact Mass (Da)** | **[M–H]⁻ *(m/z)*** | **Fragment Ions seen *(m/z)*** |
| --- | --- | --- | --- | --- |
| **TPA** | C₈H₆O₄ | 166.0266 | **165.019** | 165, 91, 211* |
| **MHET** | C₁₀H₁₀O₅ | 210.0528 | **209.045** | 209, 91, 255* |
| **MMHET**  (O-methyl-MHET) | C₁₁H₁₂O₅ | 224.0685 | **223.061** | 223+, 91, 269* |

*Fomate Adduct seen from mobile phase formic acid

+14 *m/z* due to added methyl group
